# Achieving gas pressure-dependent luminescence from an AIEgen-based metal-organic framework

**DOI:** 10.1038/s41467-022-29737-z

**Published:** 2022-04-19

**Authors:** Zhijia Li, Feilong Jiang, Muxin Yu, Shengchang Li, Lian Chen, Maochun Hong

**Affiliations:** 1 State Key Laboratory of Structure Chemistry, Fujian Institute of Research on the Structure of Matter, Chinese Academy of Sciences, Fuzhou, 350002 China; 2 University of Chinese Academy of Sciences, Beijing, 100049 China

**Keywords:** Metal-organic frameworks, Materials science

## Abstract

Materials exhibiting aggregation-induced emission (AIE) behaviour enable strong emission in solid state and can respond to various external stimuli, which may facilitate the development of materials for optical sensing, bioimaging or optoelectronic devices. Herein, we use an AIE luminogen 2’,5’-diphenyl-[1,1’:4’,1”-terphenyl]-4,4”-dicarboxylic acid as the ligand to prepare an AIEgen-based MOF (metal-organic framework) named FJI-H31. FJI-H31 exhibits bright luminescence under ambient conditions (under air and at room temperature), but almost no emission is observed under vacuum. Our investigation shows that the emission intensity displays a smooth and reversible enhancement with increased gas pressure, which may be attributed to the restriction of intramolecular motion brought by structural deformation under pressure stimulus. Unlike most pressure-responsive MOFs, the luminescence reverts to its original state once gas pressure recovers. By virtue of its unique optical properties, a luminescent MOF with sensing ability of gas-pressure is realized.

## Abstract (web summary)

Compounds displaying aggregation-induced emission behavior may have application in the preparation of smart materials. Here, the authors report a luminogen-containing metal-organic framework for which luminescence intensity changes are observed in response to gas pressure.

## Introduction

Assembled by metal ions/clusters and organic linkers, metal-organic frameworks (MOFs) are crystalline porous materials with the virtues of diverse structures, large surface areas, tunable pore sizes and versatile functionalities etc^1–4^. Taking advantage of the outstanding features of MOFs and the photoelectronic attributes from metal centers, ligands, and guests, photoluminescent metal-organic frameworks (PL-MOFs) have emerged as a promising class of stimuli-responsive luminescent materials, which are of scientific and technological interest due to their wide range of optoelectronic applications, particularly in luminescence sensing^5–8^. In the past decades, a number of PL-MOFs have been designed and synthesized for the detection of not only chemical analytes including volatile organic compounds^9^, hazard metal ions^10^, energetic materials^11^, biomarkers^12^, and dyes^13^, but also environmental changes (e.g., temperature, radiation and pH value)^14–16^. In comparison, luminescent pressure sensors based on MOFs are rarely reported. A few studies have shown that the luminescence of MOFs can respond to pressure stimulus^17–21^. Nevertheless, most of them, known as piezofluorochromism materials, display abrupt two-state switching and a phase residual after the pressure is released, failing to be employed as pressure probes^22^. Very recently, a coordination network named NKU-121 has been reported with a successive, reversible, and linear emission wavelength variation in high pressure range (1 atm to 11.12 GPa)^23^. Soon after, Zou and Shi et al. reported a tetraphenylethene based PL-MOF showing reversible piezofluorochromism from 1 atm to 11.96 GPa driven by its crystalline framework flexibility^24^. Their reversible and smooth luminescence response to pressure along with recovered phase after pressure release allow them to be promising pressure sensors. Albeit the great breakthrough of MOF-based sensors in high pressure, luminescence response in subtle-to-low pressure regime, which requires higher sensitivity of pressure-responsive materials, has not been realized in PL-MOFs heretofore, probably because it is more difficult to realize the state change of a fluorophore under small disturbance.

Conventional organic ligands often suffer from notorious aggregation caused quenching (ACQ) effect^25^, leading to weak or even no luminescence in aggregated states. Aggregation-induced emission luminogens (AIEgens), first coined by Tang and coworkers in 2001, describe a photophysical phenomenon, which emits intense luminescence in the aggregated-/solid- states while no luminescence in solution state^26–28^. Restriction of intramolecular motions (RIM) is considered as the mechanism of this unique phenomenon, which is generally categorized into restriction of intramolecular rotation (RIR) and restriction of intramolecular vibration (RIV). Anchoring AIE chromophores within MOFs not only provides a platform for further understanding the mechanism of AIE but also offers opportunities to explore attractive optical properties in new matrices. When AIEgens are coordinated to metal centers, the intramolecular vibrations or rotations are restricted, giving bright emissions with enhanced intensities and quantum yields^29^. In most cases, the phenyl rotors of AIEgens are fully substituted with coordinating groups and well rigidified in MOFs, leading to a high-level RIM^30–32^. In such highly rigid structures, the degree of intramolecular rotations is hard to regulate, which is unfavorable to the construction of stimuli-responsive luminescent materials.

In this work, we design and synthesize a half-substituted tetraphenylbenzene ligand, named [1,1′:4′,1′′-terphenyl]-4,4′′-dicarboxylic acid (H_2_TPDB), in which, only two phenyl rings of the para position are carboxylated, preserving two dangling phenyl rings whose RIR may be regulated upon external stimulus. Based on this concept, a lanthanide AIEgen-based MOF, denoted as FJI-H31, is constructed. In the rigid MOF, the phenyl rotations of the AIEgen are between the states of free rotation and complete restriction, which is reversibly fine-tuned by pressure, leading to a remarkable and continuous variation in luminescence intensity and finally realizing the gas-pressure-responsive photoluminescent MOF.

## Results

### Crystal structure

**Fig. 1:**
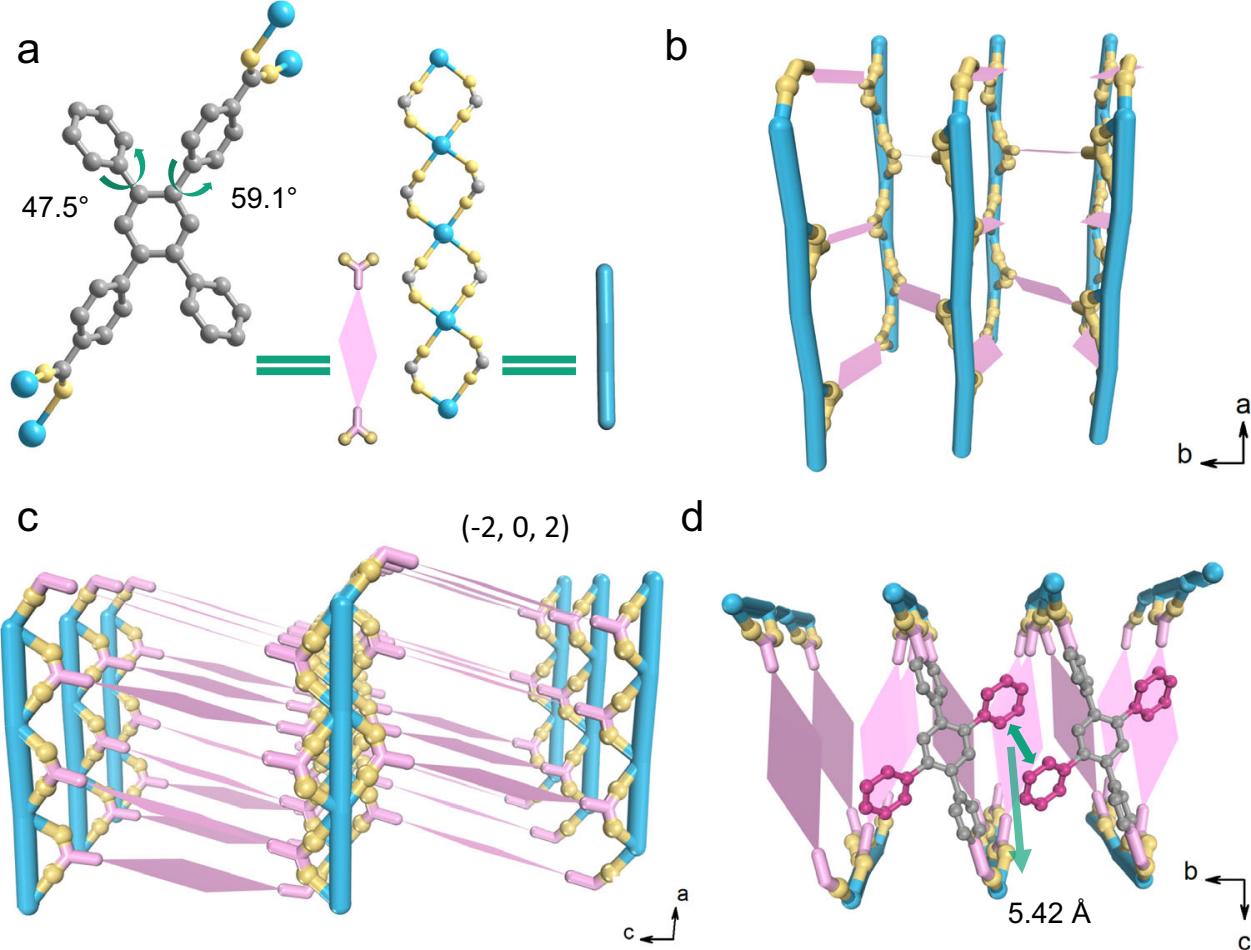
The structure of FJI-H31. **a** Left: the coordinated ligand, right: the metal 1D chain. **b** View of FJI-H31 along the *c* axis. **c** View of FJI-H31 along the *b* axis. **d** View of FJI-H31 along the *a* axis. Color code: light blue, Gd; lemon, O; sliver, C.

Colorless crystals of FJI-H31 were obtained through hydrothermal reaction of H_2_TPDB with lanthanide nitrates hexahydrate in N,N-dimethylformamide (DMF) and water at 130 °C for 3 days (see details in Supporting Information, SI). Single-crystal X-ray analysis (SCXRD) reveals that FJI-H31 crystallizes in the monoclinic space group *I*2/a with a general formula [Ln(TPDB)·2DMF$\cdot$2H_2_O]·NO_3_ (Ln = Gd^3+^, Eu^3+^). Nitrate ions can not be well defined from SCXRD but can be determined by Fourier transform infrared (FT-IR) (Supplementary Fig. 1) and are further confirmed by elemental analysis. Taking Gd compound as an example, the asymmetric unit contains a half fully deprotonated TPDB^2−^ ligand, a half crystallographically independent Gd^3+^ ion, one coordinated water molecule and one coordinated DMF molecule (Supplementary Fig. 2). The Gd^3+^ center is eight-coordinated with two oxygen atoms of DMF, two oxygen atoms of the water molecule, and four oxygen atoms of four monodentate carboxylate groups from four TPDB^2−^ ligands (Supplementary Fig. 3). Every two Gd^3+^ ions are double bridged by two carboxylic groups with a bidentate mode to extend an infinite one-dimensional metal carboxylate chain SBU (secondary building unit) (Fig. 1a). TPDB^2−^ structs interconnect these parallel arranged rod-like chains in a stagger manner (Fig. 1b), forming a 3D framework with 1D rhombus channels along the *a* axis (Supplementary Fig. 4). These channels, with the estimated dimensions of 10.53 × 35.77 Å are occupied by the pendant phenyl rings and coordinated solvents (DMF and H_2_O). The dihedral angels between central and peripheral phenyl rings of TPDB^2−^ are 59.1° and 47.5°, for carboxylated and unsubstituted phenyl rings respectively (Fig. 1a). The ligands grow along with the (−2, 0, 2) crystal face (Fig. 1c), and the closest centroid-centroid distance between dangling phenyl rings on the neighboring ligands is 5.42 Å (Fig. 1d), shorter than that in the dilute free AIEgen but longer than that in its solid state.

### Luminescence properties

**Fig. 2:**
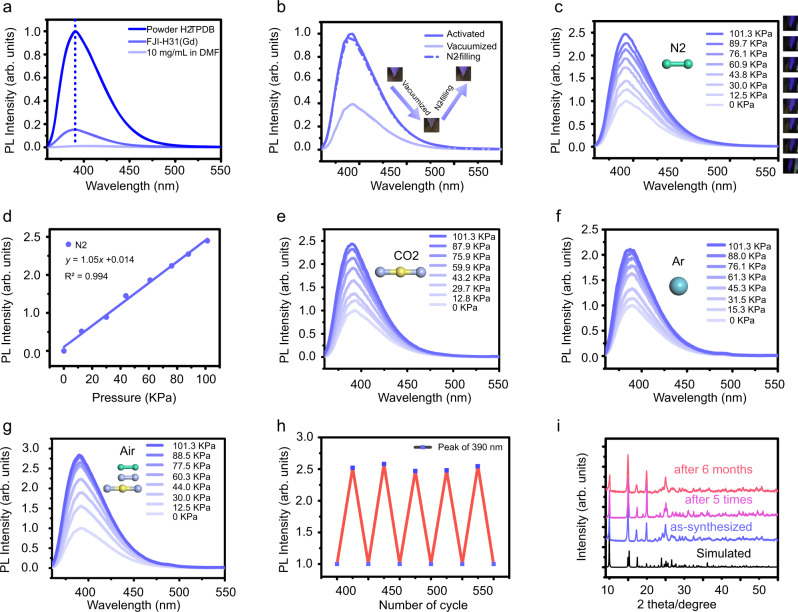
Gas-pressure-dependent luminescence properties of FJI-H31 and structural stability of FJI-H31. **a** Emission spectra of powder H_2_TPDB, FJI-H31 and the dilute solution of H_2_TPDB (10 mg/mL in DMF), excited at 338 nm. **b** Emission spectra of activated, vacuumized and N_2_-filling FJI-H31(Gd); inset: photographs of activated, vacuumized and N_2_-filling FJI-H31(Gd). **c** Emission spectra of FJI-H31(Gd) at different nitrogen (N_2_) pressures, left: the different emission color of FJI-H31(Gd) under different pressures. **d** The fitted curves for pressure-dependent intensity of FJI-H31(Gd) at 390 nm, N_2_. **e** Emission spectra of FJI-H31(Gd) at different carbon dioxide (CO_2_) pressures. **f** Emission spectra of FJI-H31(Gd) at different argon (Ar) pressures. **g** Emission spectra of FJI-H31(Gd) at different Air pressures. All spectra and photographs are excited at 338 nm, RT. **h** Reusability luminescence of FJI-H31(Gd) response to air pressure of five consecutive cycles; monitored at 390 nm. **i** Powder XRD patterns of FJI-H31: simulated, as-synthesized, after 5 times cycles and after 6 months.

**Fig. 3:**
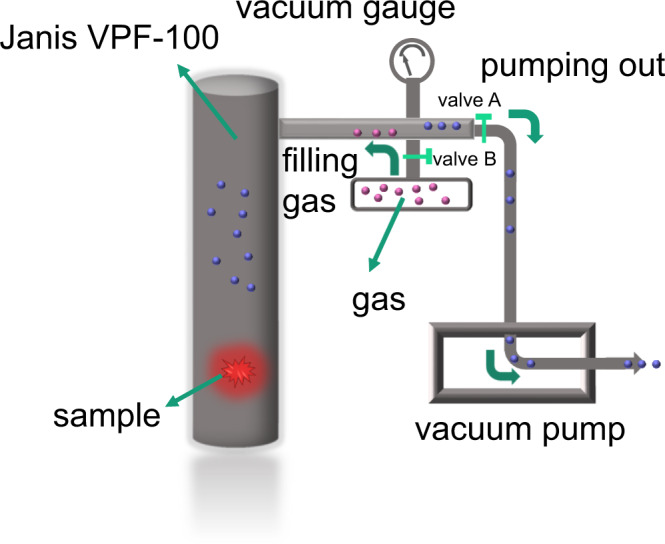
The homemade system. The illustration of a homemade gas pressure detecting equipment adapted by Janis VPF-100.

The dilute solution of H_2_TPDB (10 mg/mL in DMF) is non-emissive whereas its powdered samples exhibit a “turn-on” fluorescence centered at 390 nm (ex = 338 nm) with the absolute quantum yield (QY) as high as 53.29%, suggesting that the ligand is indeed AIE-active (Fig. 2a). To avoid the impact of metal center, Gd compound FJI-H31(Gd) was selected to study the photo-physical behaviors of the AIE fluorophore in the rigid MOF matrix. At room temperature (RT, 25 °C), FJI-H31(Gd) shows ligand-based luminescence clearly suggesting that the phenyl ring rotations/flippings of TPDB^2−^ are rigidified in the MOF. However, the intensity and the QY (3.46%) are much lower than those of the solid-state H_2_TPDB, which may mean that the restriction of intermolecular rotations is incomplete as we expected. It is noteworthy that, different from previous studies in which the emissions of AIEgens are typically blue-shifted when incorporated into a rigid MOF, the emission maximum of FJI-H31(Gd) remains almost unchanged compared with that of the solid H_2_TPDB. We ascribe this phenomenon to the unique coordination mode of the ligand. Normally, four peripheral benzene rings are all modified by the carboxyl or pyridyl groups. The coordination to metal ions in MOF lattices locks ligands in high energy conformations with larger dihedral angles between central and peripheral phenyl rings, resulting in less efficient overlap of p orbitals of the sp^2^-hybridized C atoms and thereby causing the blue-shifts of emission bands. In this study, the AIE-type strut H_2_TPDB only has two carboxylated phenyls, leaving the other two unfixed, which makes the ligand can maintain its original conformation even after coordination. Therefore, no shifts were observed for the emission peak of the ligand when anchored into the MOF matrix^33–35^. In the photoluminescence test, the activated samples (vacuumized at 120 °C for 12 h, Methods, Activation of FJI-H31) were used in order to eliminate the influence of the surrounding gas molecules. No obvious change was observed in the PXRD patterns after activation, suggesting that the activated FJI-H31(Gd) still remain its main framework and crystalline form (Supplementary Fig. 5). Then, the influence of gas pressure on the luminescence of the AIEgen-based MOF was studied. As there is no commercial equipment to support the experiment, a homemade measurement system was set up, in which, the Janis VPF-100 liquid nitrogen cooled cryostat is adapted to control and detect gas pressure. Figure 3 explains how this homemade measurement system works. Firstly, open the valve A and start the vacuum pump to pump out the air in the system. Then, close the valve A and open the valve B, at the same time, detected gas is filled step by step from gasbag. Gas pressure (vacuum degree) in the system can be real-time monitored by the vacuum gauge mounted between the Janis VPF-100 and vacuum pump. Using this homemade measurement system, the luminescence of FJI-H31(Gd) was first tested at vacuum state in which gas pressure can be considered as 0 Kpa.

As shown in Fig. 2b, fluorescence intensity shows a significant decrease at vacuum state, which is only about ca. 40% of that in ambient pressure. This means that the atmosphere does have an effect on luminescence of the AIEgen-based MOF probably by tuning the restriction of intermolecular rotations.The fluorescence can return to its original intensity when the atmosphere goes back to the normal pressure (101.3 kpa) upon inflating nitrogen. Notably, we can also observe this phenomenon by naked eyes. The luminescence of the activated FJI-H31(Gd) shows moderate lilac after outgassing treatment. Then, the emission becomes nearly invisible upon vacuuming, while recovers when exposed to nitrogen atmosphere. The recyclable test shows that the variation in luminescence intensity towards atmosphere is well reversible. It has to be mentioned that, most pressure-responsive MOFs remain phase and luminescence changes after pressure release^21,36^, while the luminescent intensity of FJI-H31(Gd) shows a quick recovery when the gas is filled.

Inspired by the above observations, the luminescence response towards different gas pressures was further investigated. After vacuumized, the surrounding gas pressure is tuned from vacuum state (0 kPa) to standard atmospheric pressure (101.3 kPa) by filling N_2_ step by step. As we expected, the intensity of ligand-centered emission rises along with the increase of the gas pressure in the whole range, reaching to about 2.5 times of the vacuum state (Fig. 2c). The luminescence spectra, showing a one-to-one correspondence between luminescence intensities and pressures, were recorded under different pressures, which is different from those measured using pressure-treated samples. There is a good linear correlation between the emission intensity and the pressure of N_2_ in the whole range (from 0 kPa to 101.3 kPa), and is fitted by the equation of *y* = 1.05*x* + 0.014 (R^2^ = 0.994) (Fig. 2d). As there is a good linear correlation between emission intensity and gas pressure which can well be fitted by the equation of *y* = *ax* + b, the slope *a* of linear equation is considered as the sensitivity of the detection. The unique pressure-dependent luminescence of FJI-H31(Gd) suggests that it may be a potential candidate of gas-pressure-responsive MOF. In order to find out whether the response of luminescence is towards the special gas molecule N_2_ or towards normal gas pressure, different kinds of gas molecules were applied to the above luminescence response test. Besides N_2_, we inflated the other three kinds of gas molecules (carbon dioxide, argon and air) into FJI-H31(Gd) and monitored the pressure-dependent luminescence intensity of the MOF. As shown in Fig. 2e–g, the luminescence of MOF manifests the similar pressure-dependent behaviors regardless of the kind of gas molecules: intensity of ligand-centered luminescence (390 nm) enhances with the filling of gases and can be well fitted by the linear equation with a slightly difference in slope (Supplementary Figs. 6–8). The obtained results show that the fluorescence intensity of the AIEgen-based MOF does respond to gas pressure rather than a special gas molecule. Benefiting from the characteristics of this unique gas-pressure-dependent luminescence, the MOF can be used as an extensive gas-pressure probe, which, as we know, has never realized in MOFs before. Moreover, the luminescence from a dark “off” state to an “on” state with the increase of the gas pressure suggests that the MOF can be employed as a turn-on type sensor which is rare in MOFs. As we know, detections based on a luminescence turn-on mechanism are highly desired because of their merits of straightforward readout, high sensitivity, selectivity and reliability.

The stability, reversibility and reusability of this material were also explored. FJI-H31(Gd) exhibits the same pressure-responsive luminescence behavior after >5 times without signal loss (Fig. 2h). Powder X-ray diffraction shows the crystallinity of FJI-H31(Gd) framework is still well maintained after multiple uses (Fig. 2i). And FJI-H31(Gd) exhibits outstanding thermal and air stabilities at the same time. The air stability test shows that FJI-H31(Gd) still maintains its original framework when exposed to the open air after more than six months. The TGA and temperature-dependent powder X-ray diffraction (TD-PXRD) were conducted to investigate the thermal stability of FJI-H31. The TGA curve reveals that the compound will be decomposed after 330 °C (Supplementary Fig. 9), and the TD-PXRD patterns show that the structure remains intact until 230 °C (Supplementary Fig. 10). These data confirm that FJI-H31 has high thermal stability. The SEM images before and after the gas pressure sensing manifest that FJI-H31 keeps intact and has no aggregate (Supplementary Figs. 11 and 12). Overall, FJI-H31(Gd) has excellent stability and reusability, making it a promising candidate for practical applications.

**Fig. 4:**
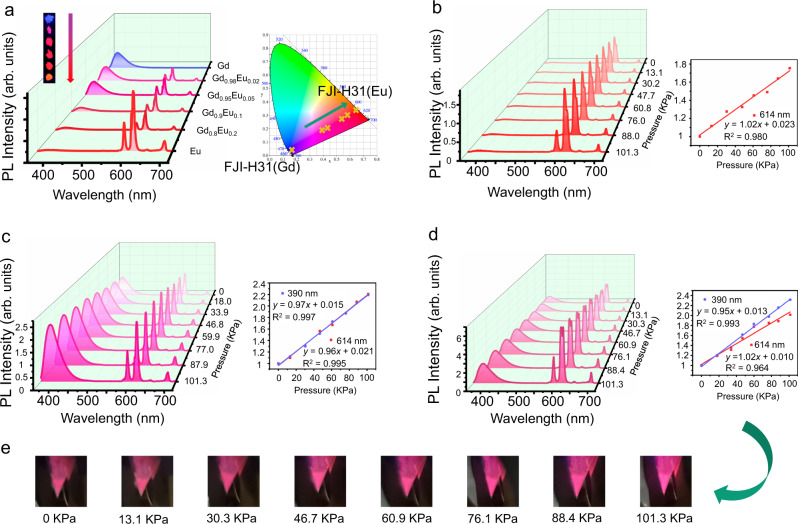
Gas-pressure-dependent luminescence properties of FJI-H31(Gd_x_Eu_1-x_). **a** Left: emission spectra of different concentrations of Gd^3+^/Eu^3+^ and photographs of different concentrations of Gd^3+^/Eu^3+^; inset, left: the different colors of FJI-H31(Gd_x_Eu_1-x_) under 365 nm ultraviolet lamp, right: the chromaticity coordinate diagram showing the luminescence color of FJI-H31(Gd_x_Eu_1-x_). **b** Left: emission spectra of FJI-H31(Eu) at different N_2_ pressures, right: the fitted curve for pressure-dependent intensity of FJI-H31(Eu) at 614 nm. **c** Left: emission spectra of FJI-H31(Gd_0.98_Eu_0.02_) at different N_2_ pressures, right: the fitted curves for pressure-dependent intensity of FJI-H31 FJI-H31(Gd_0.95_Eu_0.05_) at 614 nm. **d** Left: emission spectra of FJI-H31(Gd_0.95_Eu_0.05_) at different N_2_ pressures, right: the fitted curves for pressure-dependent intensity of FJI-H31(Gd_0.98_Eu_0.02_) at 614 nm. **e** The photographs of FJI-H31(Gd_0.95_Eu_0.05_) under UV at different pressures. All spectra are excited at 338 nm, RT.

Typical AIEgens-based MOFs exhibit single emission ascribed to the ligand. Antenna effect is an important luminescence behavior which is often observed from lanthanide coordination complexes. Direct excitation of Ln^3+^ 4 f electron transitions is prohibitively inefficient since the 4f-4f electron transitions are Laporte forbidden. Organic ligand can act as “antenna” which absorbs photons and transfer the energy to the nearby Ln^3+^, producing a bright luminescence^37,38^. Take advantage of antenna effect, we can change the emission wavelength of a lanthanide metal-organic framework (LnMOF) by introducing different Ln ions or obtain the dual emissions upon single excitation by a mixed-lanthanide framework, giving a chance to fabricate luminescent sensors with desired emission colors or dual emissions. The phosphorescence spectrum of compound FJI-H31(Gd) at 77 K reveals that the triplet state energy level T_1_ of the AIEgen is 21,739 cm^−1^ (460 nm) (Supplementary Fig. 13), suggesting it is an excellent antenna chromophore for sensitizing the europium ions (17,267 cm^−1^) (Supplementary Figs. 14 and 15). When Gd^3+^ is replaced by Eu^3+^, the obtained FJI-H31(Eu) displays a red luminescence with characteristic emission lines of Eu^3+^ ions at 591, 614, 650, and 699 nm (^5^D_0_ → ^7^F_J_, J = 1, 2, 3 and 4) (excited at 338 nm). The disappearance of ligand-centered emission (390 nm) manifests the efficient sensitization from AIEgen to europium ions. In this case, the energy absorbed by the ligand is well transferred to the lanthanide ion, successfully tuning the emission color of the MOF from purple (390 nm) to red (614 nm). If the energy transfer is incomplete, both ligand- and metal-centered emissions could be obtained at the same time, endowing the luminescent MOF with the dual emissions. To this end, a series of bimetallic lanthanide MOFs were synthesized by incorporating different molar ratios of Gd^3+^ and Eu^3+^ ions into the framework. As shown in Fig. 4a, both ligand- and metal-centered emissions (peaked at 390 nm and 614 nm respectively, ex = 338 nm) can be observed as the concentration of Gd^3+^ changes and the relative intensity of the two emissions can be tuned by the proportions of the lanthanide ions, showing different luminescence colors (the actual molar ratios of Gd/Eu determined by ICP are shown in Supplementary Table 2). When the concentration of Eu^3+^ is increased to ca. 2%, two emissions are comparable. The further doping of Eu^3+^ makes the metal-centered emission stronger while the ligand-centered emission weaker, and finally disappears. The lifetimes and QYs of mixed LnMOFs and H_2_TPDB are measured and listed in Supplementary Table 3 and Supplementary Fig. 16–19.

As mentioned above, we can change the emission color of the LnMOF by metal replacement or obtain dual emission by mixed-metal strategy. Gas-pressure responsive experiments were then conducted to find out if the unique luminescence property has been preserved in the obtained materials. As Fig. 4b shown, emission peak of FJI-H31(Eu) at 614 nm manifests similar turn-on gas-pressure-dependent behavior just like that of FJI-H31(Gd) at 390 nm. The intensity of peak 614 nm also shows a good relationship with gas pressure and the curve can be well fitted by the equation *y* = 1.02*x* + 0.023 (R^2^ = 0.980). Despite of the energy transfer from AIEgen to lanthanide ions, the slope of the linear curve, which reflects the sensitivity of the detection, have little difference with that in FJI-H31(Gd). For mixed LnMOF, FJI-H31(Gd_0.95_Eu_0.05_) and FJI-H31(Gd_0.98_Eu_0.02_), both the ligand-centered and metal-centered emissions display similar gas-pressure response as observed in FJI-H31(Gd) and FJI-H31(Eu). Slopes for linear curves corresponding to two emissions are almost the same in FJI-H31(Gd_0.98_Eu_0.02_) while show a little deference in FJI-H31(Gd_0.95_Eu_0.05_). (Fig. 4c, d). Luminescence change of FJI-H31(Gd_0.95_Eu_0.05_) under different pressures can be viewed as Fig. 4e shown. Luminescent materials possessing dual emissions offer the opportunities to fabricate sensors with high reliability, accuracy or self-calibration^39,40^.

### Detection mechanism

**Fig. 5:**
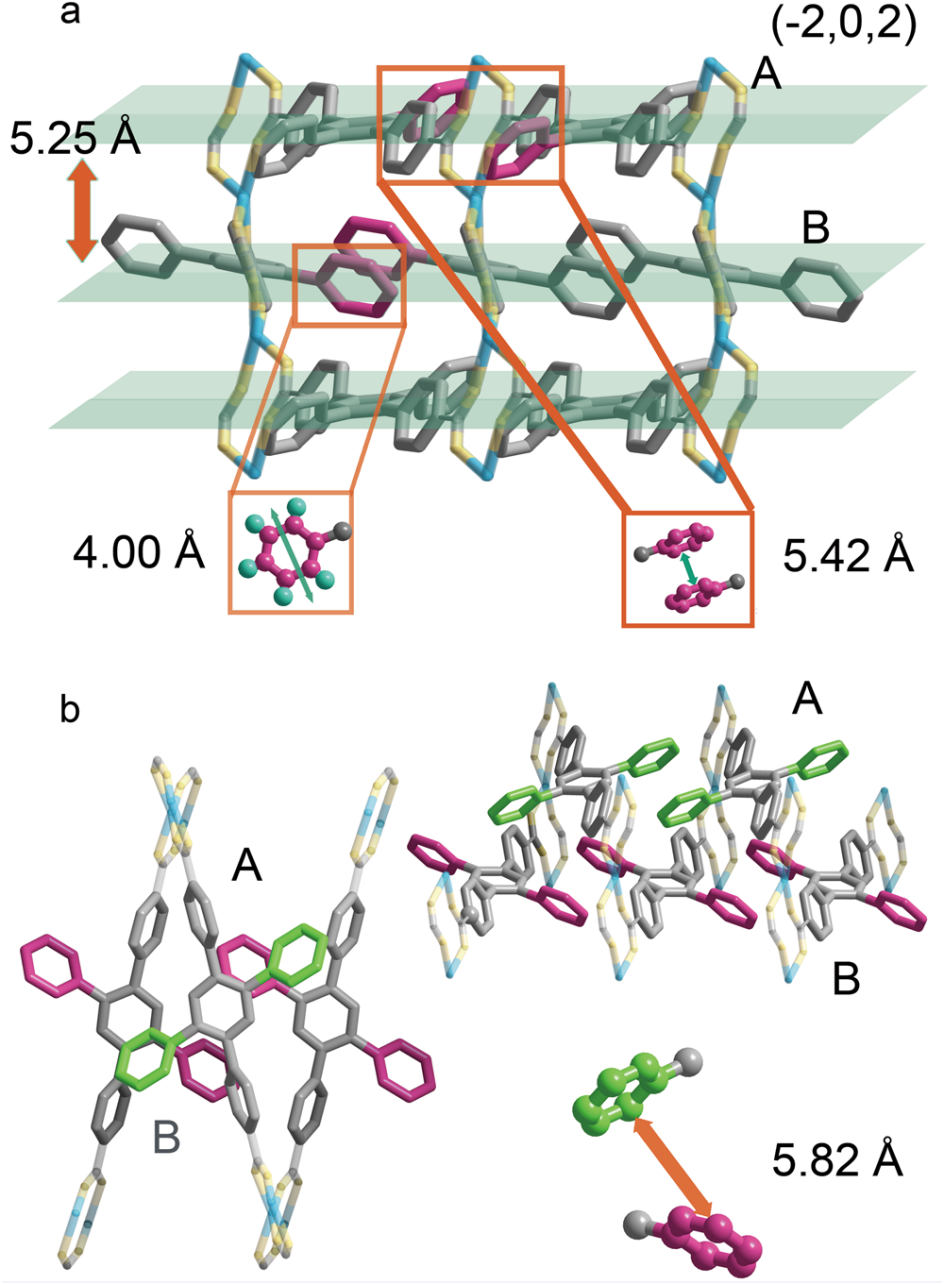
Analysis of the structure of FJI-H31. **a** The distance between each paralleled ligand along with the (−2, 0, 2) face, the distance of adjacent ligands and the distance of rotating diameter**. b** The ABAB stacking mode of (−2, 0, 2) face.

To explore the underlying mechanism of the extraordinary luminescence response induced by gas pressure, we look into the crystal structure of the AIEgen-based MOF in detail. Look along the *c* direction (Fig. 5a), the distance between the two paralleled (−2, 0, 2) crystal face is 5.25 Å, and layers are staggered in an ABAB mode (Fig. 5b). The closest dangling phenyls in neighboring layers are not parallel with a long centroid-centroid distance (5.82 Å), failing to form interactions (π-π or RIM) with each other (Fig. 5b). In contrast, the unsubstituted phenyls in the same layer are paralleled with the shorter distance of 5.42 Å, and therefore are supposed to be a key factor influencing the restriction of intermolecular motions. Considering that the diameter of the phenyls is 4.00 Å, these phenyl rings can rotate in a certain degree but not freely^41^. Thus, the phenyl rotations of TPDB^2−^ are between the states of free rotation and complete restriction, which affords opportunities for responses towards external stimuli. This is also verified by the fact that the luminescence of FJI-H31 is very weak compared with that of the aggregated AIEgen. As a consequence, FJI-H31 has an extremely sensitive luminescence for external stimuli.

**Fig. 6:**
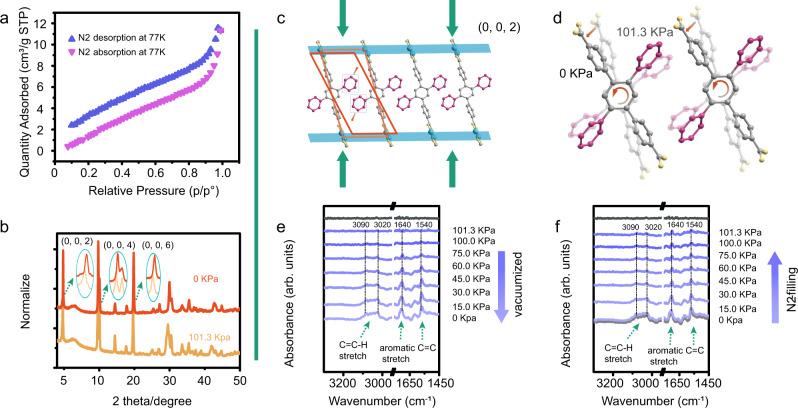
Detection mechanism of FJI-H31. **a** The N_2_ adsorption-desorption isotherms of FJI-H31(Gd) at 77 K. **b** The PXRD patterns of FJI-H31(Gd) at normal atmosphere and vacuum state. **c** The shrunken (0, 0, 2) face after vacuumized. **d** The proposed change of dangling phenyls. **e** The in situ FT-IR spectra recorded at different pressures during vacuumization, showing that the C-H stretching vibration and aromatic C=C stretching vibration become stronger, **f** while recover to originally upon filling N_2_, RT.

N_2_ was applied to adsorption test to invest the pore channel. The typical adsorption-desorption isotherms reveal that N_2_ absorption of FJI-H31 is only about 11.3 cm³/g at 1.01 bar (77 K) (Fig. 6a), and the Brunauer–Emmett–Teller (BET) surface area is calculated to be 2.29 m²/g. The results indicate that gas molecules cannot enter the channels of the MOF, probably because the suspended aromatic rings and coordinated solvents occupy the space of channels. To further confirm this conclusion, CO_2_ adsorption-desorption isotherms under 298 K, 1.01 bar were also recorded (Supplementary Fig. 20). The result shows that CO_2_ absorption of FJI-H31 is only about 12.47 cm³/g at room temperature, suggesting that CO_2_ molecules cannot enter the channels of the FJI-H31 either. The simulation of the pore size distributions of FJI-H31 with Zeo ++  shows that this structure has a 2.26 Å GCD (global cavity diameter), 1.28 Å PLD (pore limiting diameter), and 2.26 Å LCD (largest cavity diameter)^42^, smaller than most of gas molecules. The results are consistent with that of N_2_ adsorption-desorption experiment. Thus, the mechanism that the gas molecules ingress the cavity of the MOF to directly restrict the rotations of dangling benzene rings can be ruled out. Meanwhile, PXRD patterns of FJI-H31(Gd) at vacuum state (0 kpa) were recorded and compared with those measured at normal atmosphere (101.3 kpa). As shown in Fig. 6b, PXRD patterns under different gas pressures are quite similar on the whole. A closer look shows that the diffraction peak of (0, 0, 2) crystal face at vacuum state (0 kpa) shifts to the right by about 0.03°, implying a shorter distance between these crystal faces^43^. This subtle change in structure can also be further corroborated by the right shifts of the related (0, 0, 4) and (0, 0, 6) faces, by ca. 0.05° and 0.09° respectively. Based on above information, we assume that the small deformation of crystal structure may be mainly responsible for the unique gas-pressure-dependent luminescence of the MOF. A possible mechanism is proposed as illustrated in Fig. 6c, d: as pressure decreases, the AIE-gens in the same layer rotate anticlockwise by a certain angle, leading a further separation of the neighboring suspended benzene rings, which brings less restriction of intermolecular rotations, a higher rate of the nonradiative decay, and finally the diminishment of fluorescence intensity. Here, the MOF offers an appropriate matrix so that the RIR of the AIEgen can be tuned by gas pressure for luminescence response. This can be further supported by the fact that the luminescence of pure H_2_TPDB in solid state shows no response to gas pressure (Supplementary Fig. 21), probably because its compact structure cannot provide enough space for the regulation of RIR.

Although the PXRD manifests that the diffraction peaks shift right after vacuumized, it is lacking of evidence that the crystal faces keep away from each other slowly. In-situ FT-IR can monitor the vibrations of different groups in real-time, thus can disclose whether this variation is continuous or intermittent. First, air was pumped out step by step and FT-IR spectra were recorded under different pressures (Fig. 6e). As gas pressure decreases, the bands ascribed to aromatic C-H stretching vibration (3020–3090 cm^−1^) and aromatic C=C stretching vibration (1540 and 1640 cm^−1^) grow slowly, indicating the more active molecular motion of the phenyl rings. After vacuumized, N_2_ was filled in, tuning the surrounding pressure from 0 Kpa to 100.0 Kpa gradually. As Fig. 6f shown, the above IR bands abate with the recovery of the gas pressure. The reversible change in IR spectra is well consistent with the luminescence response towards gas pressure, implying molecular motion of the phenyl rings is the main factor for gas-pressure-dependent luminescence. Exhausting air and filling N_2_ only bring about change in the intensity of the characteristic bands. No shifts were observed in the in situ FT-IR spectra. That means gas molecules did not go in or out of the MOF and interact with the phenyl rings. Thus, the restriction of molecular motion does not come from the direct interaction between gas molecules and the MOF. Reversible structural deformation induced by gas pressure, which tunes the intramolecular space for RIM, is a more likely explanation.

## Discussion

In summary, a luminescent MOF (FJI-H31) has been synthesized by employing a half-substituted AIE luminogen H_2_TPDB as the ligand. The MOF shows reversible gas-pressure-dependent luminescence which can return to its original state with the recovery of the pressure, achieving the luminescent MOF with gas-pressure sensing ability based on MOFs. The material displays excellent stability, universality and reusability, and its luminescence color can be tuned by making use of antenna effect of lanthanide ions, giving a chance for practically industrial applications in the future. We consider that the unique state of AIEgen ligand in FJI-H31 may be mainly responsible for this gas pressure sensing ability. In FJI-H31, the rotation of the dangling phenyl rings of the TPDB^2−^ is between the states of free rotation and complete restriction. The special state of the AIEgen endowed by MOF offers the opportunity that the intramolecular rotation can be regulated by gas pressure and thus affect the luminescence intensity of the MOF. This work demonstrates the utilization of the guest molecules to control the degree of intramolecular rotations for sensing, which is a promising way to explore sensors for gas pressure and obtain a better understanding of the mechanism of aggregation-induced emission. Furthermore, the research encourages us to explore more turn-on sensors by incorporating AIEgens to MOF.

## Methods

### Synthesis of 2’,5’-dibromo-1,1’:4’,1”-terphenyl

The synthesis of 2′,5′-dibromo-1,1′:4′,1′′-terphenyl was followed the previous report with some modifications^44^. 1,4-dibromo-2,5-diiodobenzene (2.927 g, 6 mmol, 1.0 equiv.), phenylboronic acid (1.902 g, 15.6 mmol, 2.6 equiv.), potassium carbonate (3.317 g, 24 mmol, 4.0 equiv.), 1,4-dioxane (120 mL), tetrakis (triphenylphosphine) palladium (0.139 g, 0.12 mmol, 5 mol%) were added to a 250 mL schlenk flask charged with stir bar. The flask was pumped under vacuum and refilled with Ar for three times, and then 120 mL degassed 1,4-dioxane was transferred to the system and the reaction mixture was heated to 85 °C for 72 h under Ar atmosphere. Upon completion, the reaction was diluted with water, extracted 3 times with DCM, dried on MgSO_4_, and concentrated via rotary evaporation. The solid was then recrystallized in EtOH and provided 1.065 g (2.7 mmol, 45% yield) of white needle-like crystals. ^1^H NMR (400 MHz, CDCl3): δ 7.65 (s, 2H), 7.47-7.42 (m, 10H) (Supplementary Fig. 22).

### Synthesis of 2’,5’-diphenyl-[1,1’:4’,1”-terphenyl]-4,4”-dicarboxylic acid (H_2_TPDB)

The synthetic route of the ligand [2′,5′-diphenyl-[1,1′:4′,1′′-terphenyl]-4,4′′-dicarboxylic acid (H_2_TPDB) is shown in Supplementary Fig. 23. 2′,5′-dibromo-1,1′:4′,1″-terphenyl (0.776 g, 2 mmol, 1.0 equiv.), (4-(methoxycarbonyl)phenyl) boronic acid (1.080 g, 6 mmol, 3.0 equiv.), K_2_CO_3_ (1.106 g, 8 mmol, 4.0 equiv.) and tetrakis(triphenylphosphine)palladium (0.139 g, 0.12 mmol, 6 mol%) were added to a 250 mL schlenk flask charged with stir bar. The flask was pumped under vacuum and refilled with Ar for three times, and then 120 mL degassed 1,4-dioxane was transferred to the system and the reaction mixture was heated to 85 °C for 72 h under Ar atmosphere. After the reaction mixture was cooled to room temperature, the organic solvent was removed using rotary evaporator, and the resulting mixture was poured into water and extracted with dichloromethane (3 × 100 mL). The combined organic layers were dried over anhydrous MgSO_4_, and then the solvent was removed again using rotary evaporator. After purification by column chromatography on silica gel using dichloromethane/ petroleum ether as eluent and evaporation of the fraction containing the product, compound dimethyl 2′,5′-diphenyl-[1,1′:4′,1′′-terphenyl]-4,4′′-dicarboxylate was obtained as white solid. Then it was dissolved in 30 mL of THF, to which 50 mL of 10 M NaOH aqueous solution was added. The mixture was stirred under reflux for 10 h, and then the organic solvent was removed using rotary evaporator. The aqueous phase was acidified to pH 2 using 6 M HCl aqueous solution. The resulting precipitate was collected via filtration, washed with water (200 mL), and dried under vacuum to afford 0.827 g (1.7 mmol, 85% yield) H_2_TPDB. ^1^H NMR (400 MHz, DMSO-d6) δ, 7.21–7.32 (m, 10H), 7.32–7.38 (d, 4H), 7.48–7.53 (s, 2H), 7.80–7.87 (d, 4H), 12.90–13.05 (s, 2H) ppm (Supplementary Fig. 24).

### Synthesis of FJI-H31(Gd)/ FJI-H31(Eu)

A mixture of Ln(NO_3_)_3_·6H_2_O (5.6 mg, 0.0125 mmol) and 2′,5′-diphenyl-[1,1′:4′,1′′-terphenyl]-4,4′′-dicarboxylic acid (H_2_TPDB) were dissolved in DMF (N,N-dimethylformamide) (2 mL), H_2_O (1 mL) and HCl (50 μL) with ultrasonic, then sealed in a 25 mL Teflon chamber within a Teflon-lined stainless steel autoclave and heated at 130 °C for three days. Colorless crystals FJI-H31 were obtained after the mixture was slowly cooled to room temperature, washed with DMF and MeOH, and dried in air. The yields are ca. 58.6% and 61.7% for FJI-H31(Eu) and FJI-H31(Gd) based on the ligand, respectively. The phase purities of all compounds were confirmed by comparison of powder X-ray diffraction patterns with data collected from the single crystal diffraction (Supplementary Fig. 25)^45–47^. Elemental analysis (%): Calcd (%) for GdC_38_H_38_N_3_O_11_ (Mr = 869.98): C, 52.46; H, 4.40; N, 4.83; O, 20.23. Found (%): C, 53.40; H, 4.19; N, 4.57; O, 20.60. Calcd (%) for EuC_38_H_38_N_3_O_11_ (Mr = 864.69): C, 52.78; H, 4.43; N, 4.86; O, 20.35. Found (%): C, 53.65; H, 4.24; N, 4.76; O, 20.83.

### Synthesis of M’LnMOFs

The Gd/Eu mixed MOFs FJI-H31(Gd_1−x_Eu_x_) (x = 0.2, 0.1, 0.5, 0.02, 0.005) were synthesized similarly to FJI-H31(Gd) except for the use of a mixture of Gd(NO_3_)_3_·6H_2_O and Eu(NO_3_)_3_·6H_2_O as starting materials. The PXRD patterns indicate that all mixed lanthanide MOFs are isostructural to the parent ones FJI-H31(Gd) and FJI-H31(Eu) (Supplementary Fig. 26). The molar ratios of Gd/Eu in such mixed lanthanide MOFs were determined by the ICP analyses, and the ICP samples were prepared by digesting the dry samples into concentrated HCl (37%) (Supplementary Table 2).

### Activation of FJI-H31

10 mg FJI-H31 washed by DMF and MeOH each three times. Then the powder was activated by thermal activation at 120 °C under vacuum for 12 hours to prepare activated FJI-H31.

### Materials

All relevant chemicals and solvents were reagent-grade quality and used in the synthetic processes as commercially purchased without any further purification.

### Characterization

All the crystal structure data of compounds were collected on a SuperNova diffractometer equipped with a Multilayers mirror Cu Kα radiation (λ = 1.54 Å) by using a ω scan. Powder X-ray diffraction (PXRD) patterns were collected with a Rigaku MiniFlex 600 diffractometer using Cu *K*α radiation (*λ* = 1.54 Å). The variable-temperature PXRD patterns were collected by a Rigaku Smartlab 3KW X-ray diffractometer using Cu *K*α radiation (*λ* = 1.54 Å) in N_2_ atmosphere. The vacuumed PXRD patterns were collected by Rigaku SmartLab SE and Anton Paar XRK900 using Cu Kα radiation (*λ* = 1.54 Å) at room temperature. FT-IR spectra were obtained on Bruker VERTEX 70 FT-IR Spectrometer. In situ FT-IR spectra were obtained on Thermo Nicolet 6700 FT-IR Spectrometer. ^1^H NMR spectra were obtained on a Burker AVANCE 400 (400 MHz) for spectrometer. Elemental analyses (C, H, N and O) were performed on a German Elementary Vario EL III instrument. Thermogravimetric analyses (TGA) were recorded in the temperature range of 30–900 °C, with a heating rate of 10 °C·min^−1^ under a flowing nitrogen atmosphere on a Netzsch Model STA 449 C instrument. Emission and excitation spectra in the solid state were acquired on a Horiba Jobin-Yvon Fluorolog-3 spectrofluorometer with Janis VPF-100 liquid nitrogen cooled cryostat. The 77 K phosphorescence were carried out on a FLS1000 spectrofluorometer with a continuous xenon lamp (450 W). The N_2_ adsorption/desorption isotherms were recorded at ASAP 2020 M. The overall photoluminescence quantum yields were obtained on an integrating sphere covered with barium sulfate at room temperature.

## Supplementary information

Supplementary Information

## Data Availability

The data that support the findings of this study are available in the article or its Supplementary Information File, or available from the corresponding author on request. The X-ray crystallographic coordinates for structures reported in this study have been deposited at the Cambridge Crystallographic Data Centre (CCDC), under deposition numbers 2041736 and 2041737. These data can be obtained free of charge from The Cambridge Crystallographic Data Centre via www.ccdc.cam.ac.uk/data_request/cif.
