# Supplementary material for: Achieving gas pressure-dependent luminescence from an AIEgen-based metal-organic framework
Source: Nat Commun. 2022 Apr 19;13:2142. doi: 10.1038/s41467-022-29737-z (PMC9018843; doi:10.1038/s41467-022-29737-z)
Supplement: Supplementary file 1 — Supplementary Information [file 41467_2022_29737_MOESM1_ESM.pdf]

Supplementary Information for

Achieving Gas-Pressure-Dependent Luminescence from an AIEgen-Based Metal-Organic Framework

Zhijia Li[1, 2], Feilong Jiang[1], Muxin Yu[1], Shengchang Li[1], Lian Chen[*, 1], Maochun Hong[*, 1]

[1]State Key Laboratory of Structure Chemistry, Fujian Institute of Research on the Structure of Matter, Chinese Academy of Sciences, Fuzhou 350002, China

[2]University of Chinese Academy of Sciences, Beijing, 100049, China
Email: cl@fjirsm.ac.cn; hmc@fjirsm.ac.cn

Contents

1.Supplementary Figures

Supplementary Figure 1. The FT-IR spectrum of FJI-H31(Gd). 1410 cm^{-1} suggesting the existence of nitrate ions.
Supplementary Figure 2. The asymmetric unit of FJI-H31(Gd).
Supplementary Figure 3. The coordination environment of Gd^{3+}.
Supplementary Figure 4. View of FJI-H31(Gd) along the *a* axis. Color code: green, Gd; red, O; sliver, C; bule, N; white, H.
Supplementary Figure 5. Powder XRD patterns of freshly prepared and activated FJI-H31(Gd).
Supplementary Figure 6. The fitted curve for pressure-dependent intensity of FJI-H31(Gd) at 390 nm, CO_2.
Supplementary Figure 7. The fitted curve for pressure-dependent intensity of FJI-H31(Gd) at 390 nm, Ar.
Supplementary Figure 8. The fitted curve for pressure-dependent intensity of FJI-H31(Gd) at 390 nm, Air.
Supplementary Figure 9. The curve of the thermogravimetric analysis of FJI-H31(Gd). FJI-H31 will decompose after 330 ℃.
Supplementary Figure 10. Powder XRD patterns of FJI-H31 at different temperatures, compared with simulated FJI-H31.
Supplementary Figure 11. SEM images of FJI-H31 before gas pressure sensing.
Supplementary Figure 12. SEM images of FJI-H31 after gas pressure sensing.
Supplementary Figure 13. Phosphorescence spectrum of FJI-H31(Gd) at 77 K.
Supplementary Figure 14. Excitation spectrum of FJI-H31(Eu), monitored at 614 nm.
Supplementary Figure 15. Excitation spectrum of FJI-H31(Gd), monitored at 390 nm.
Supplementary Figure 16. The luminescence decay curve of H_2TPDB at 390 nm.
Supplementary Figure 17. The luminescence decay curve of FJI-H31(Eu) at 614 nm.
Supplementary Figure 18. The luminescence decay curve of FJI-H31($Gd_{0.98}Eu_{0.02}$) at 614 nm.
Supplementary Figure 19. The luminescence decay curves of FJI-H31($Gd_{0.95}Eu_{0.05}$) at 614 nm.
Supplementary Figure 20. The CO_2 adsorption-desorption isotherms of FJI-H31(Gd) at 298K.
Supplementary Figure 21. Emission spectra of H_2TPDB before and after vacuumized, excited at 338 nm.
Supplementary Figure 22. The ^{1}H-NMR spectrum of 2',5'-dibromo-1,1':4',1"-terphenyl (400 MHz, $CDCl_3$, 298 K).
Supplementary Figure 23. Synthesis scheme of the ligand H_2TPDB. Firstly, synthesis of 2',5'-dibromo-1,1':4',1"-terphenyl by 1,4-dibromo-2,5-diiodobenzene and phenylboronic acid; then this product was mixed with (4-(methoxycarbonyl)phenyl) boronic acid to synthesize dimethyl 2',5'-diphenyl-[1,1':4',1"-terphenyl]-4,4"-

dicarboxylate; finally, the carboxylic ester was hydrolyzed to get the target product H_2TPDB.
Supplementary Figure 24. The ^{1}H-NMR spectrum of H_2TPDB (400 MHz, DMSO-D_6, 298 K).
Supplementary Figure 25. Powder XRD patterns of synthesized FJI-H31(Gd) and FJI-H31(Eu).
Supplementary Figure 26. Powder XRD patterns of samples with different ratios of Gd^{3+}/Eu^{3+}.
Supplementary Table 1. Crystal data and structure refinement parameters for FJI-H31(Gd) and FJI-H31(Eu).
Supplementary Table 2. The original ratios of different lanthanide metal salts and the corresponding ICP results.
Supplementary Table 3. The lifetimes and QYs of H_2TPDB and lanthanide-MOFs with different molar ratios of Gd^{3+} and Eu^{3+}.

1. Supplementary Figures

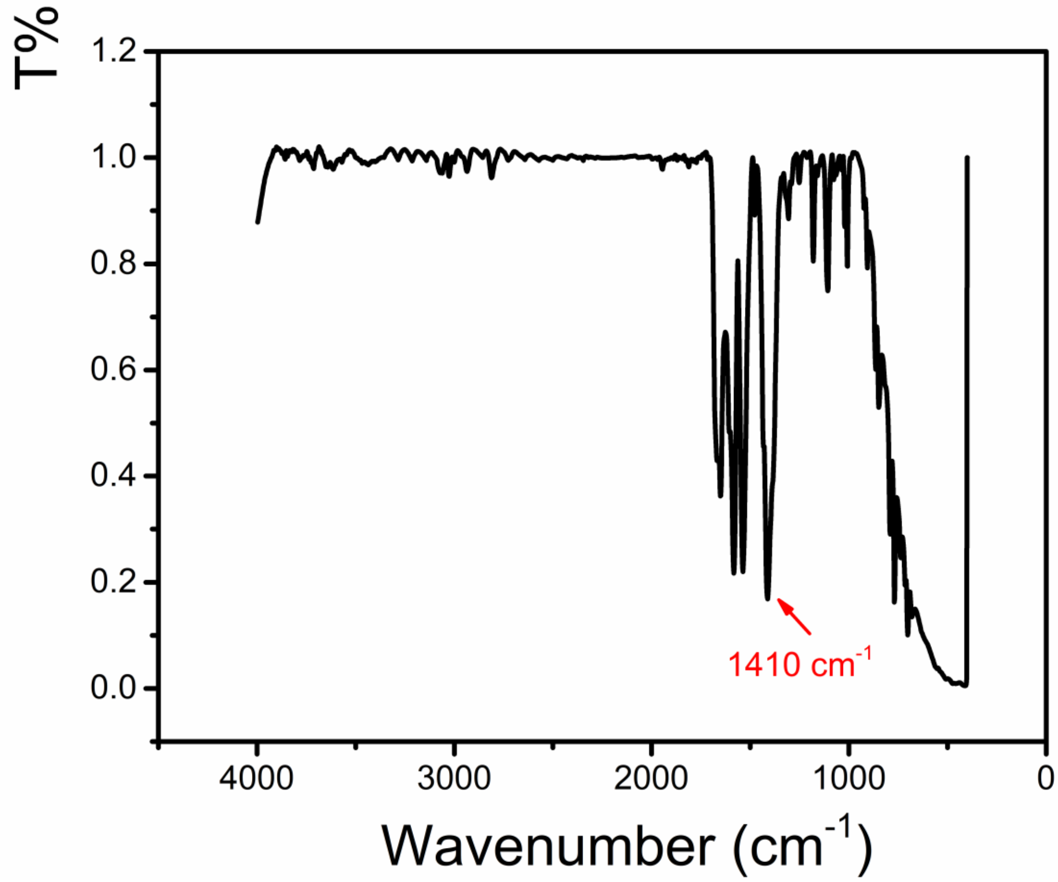

Supplementary Figure 1. The FT-IR spectrum of FJI-H31(Gd). 1410 cm^{-1} suggesting the existence of nitrate ions.

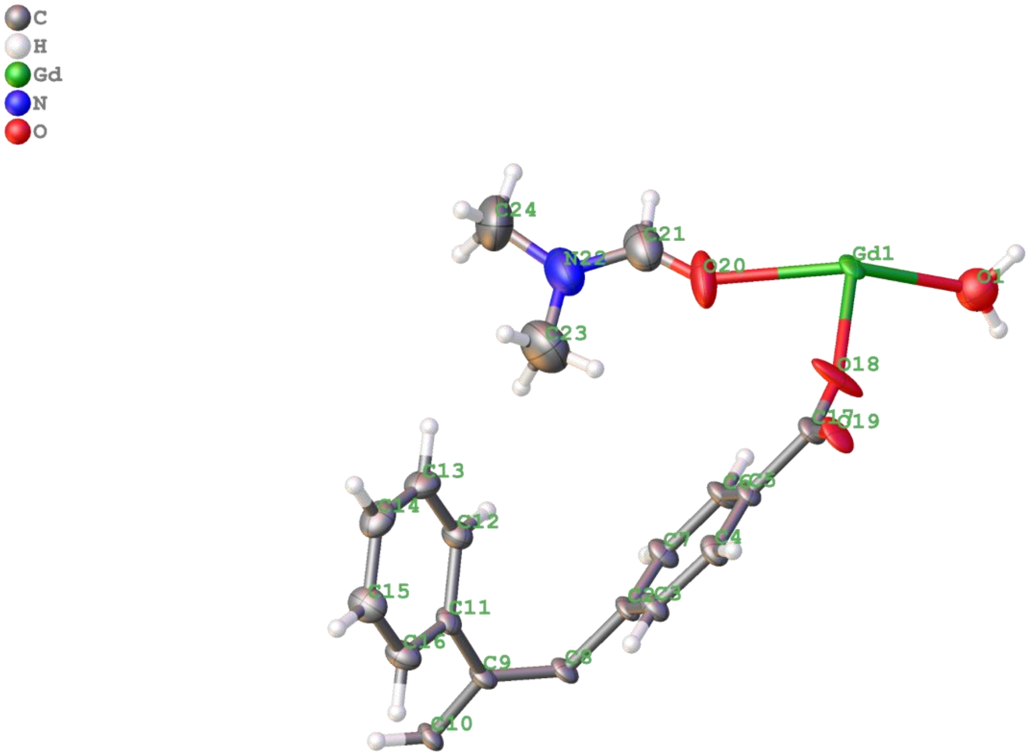

Supplementary Figure 2. The asymmetric unit of FJI-H31(Gd).

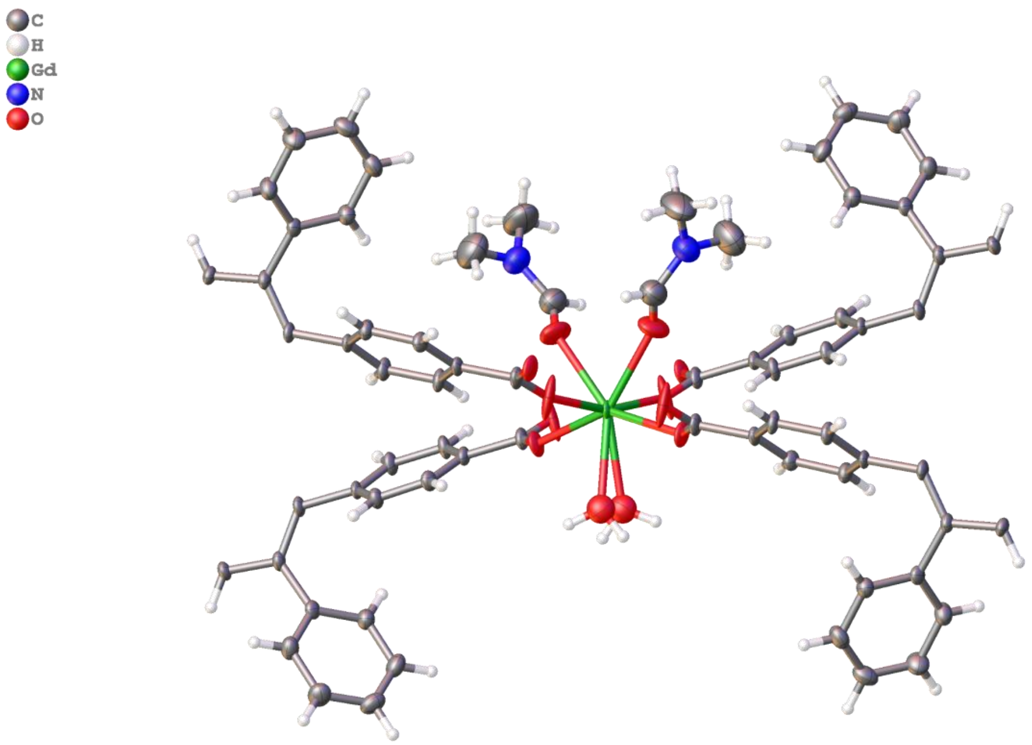

Supplementary Figure 3. The coordination environment of Gd^{3+}.

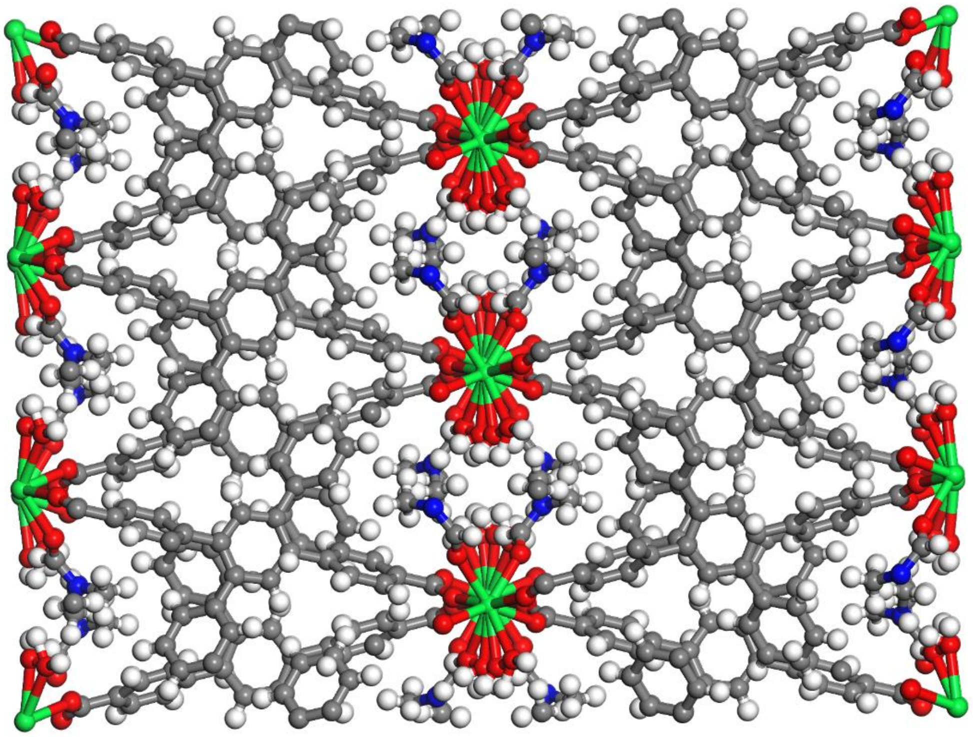

Supplementary Figure 4. View of FJI-H31(Gd) along the *a* axis. Color code: green, Gd; red, O; sliver, C; bule, N; white, H.

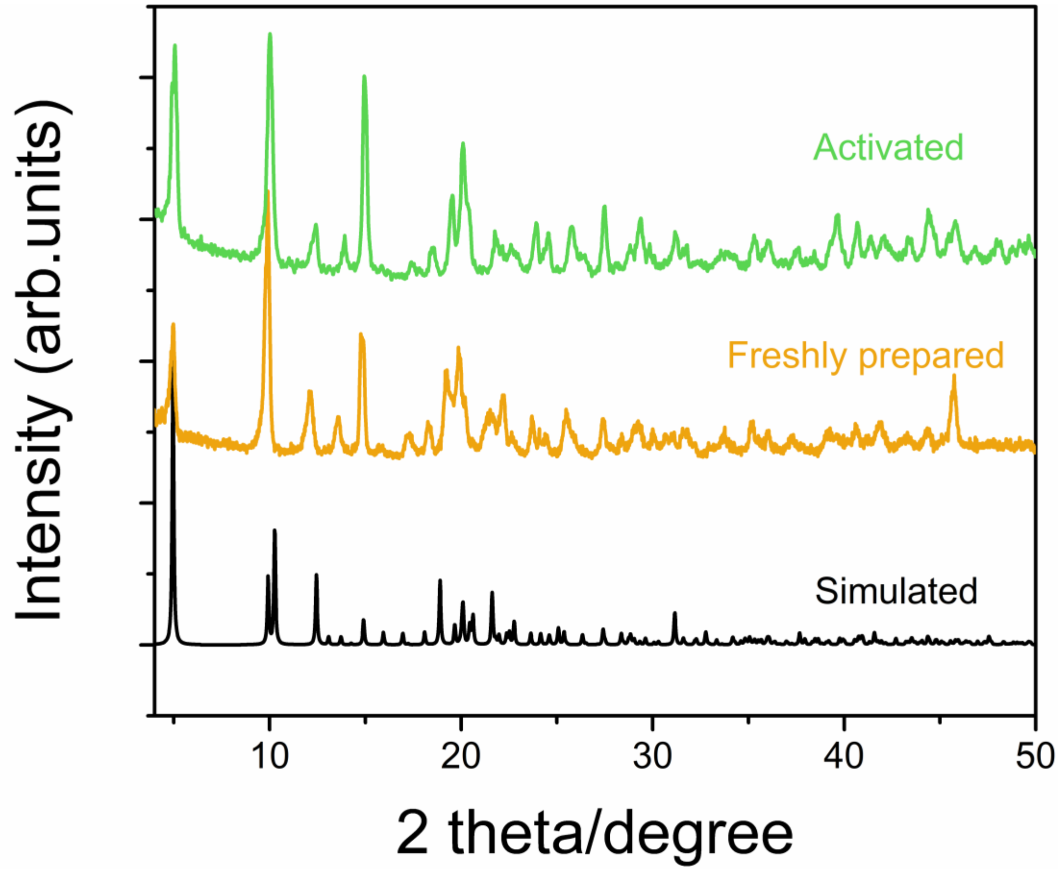

Supplementary Figure 5. Powder XRD patterns of freshly prepared and activated FJI-H31(Gd).

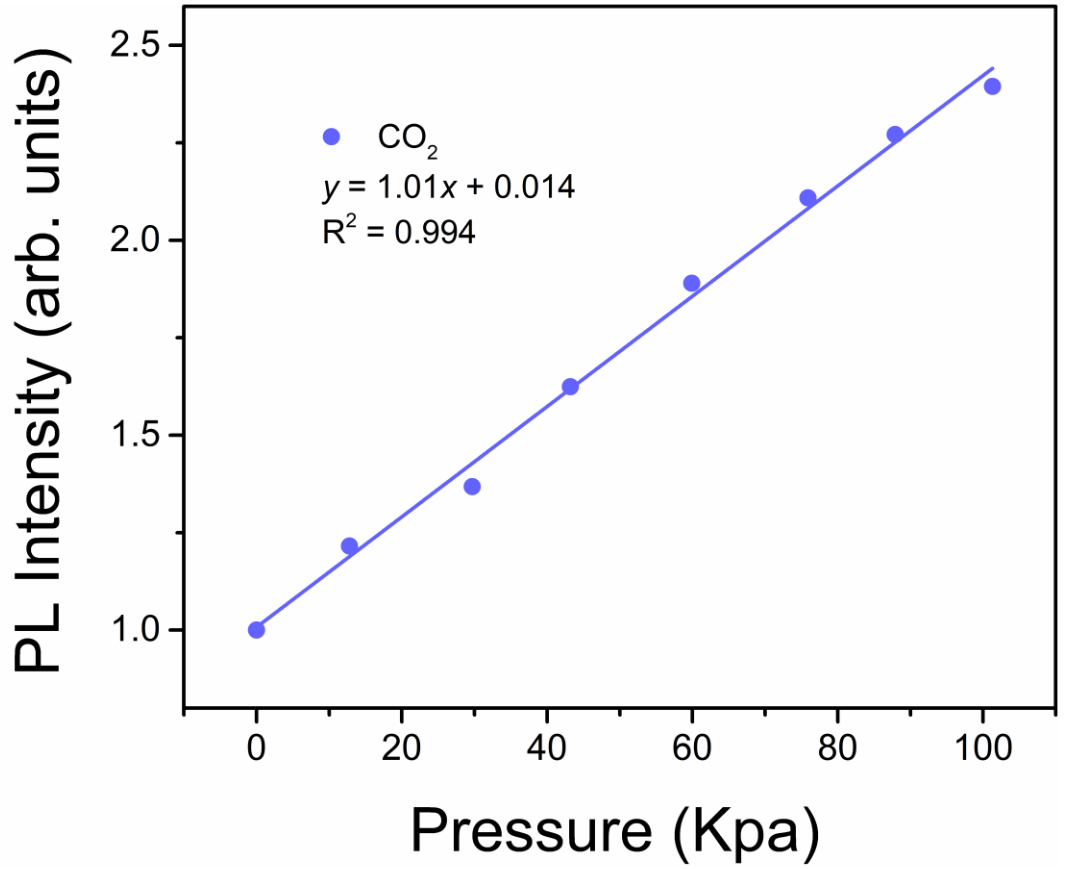

Supplementary Figure 6. The fitted curve for pressure-dependent intensity of FJI-H31(Gd) at 390 nm, CO_2.

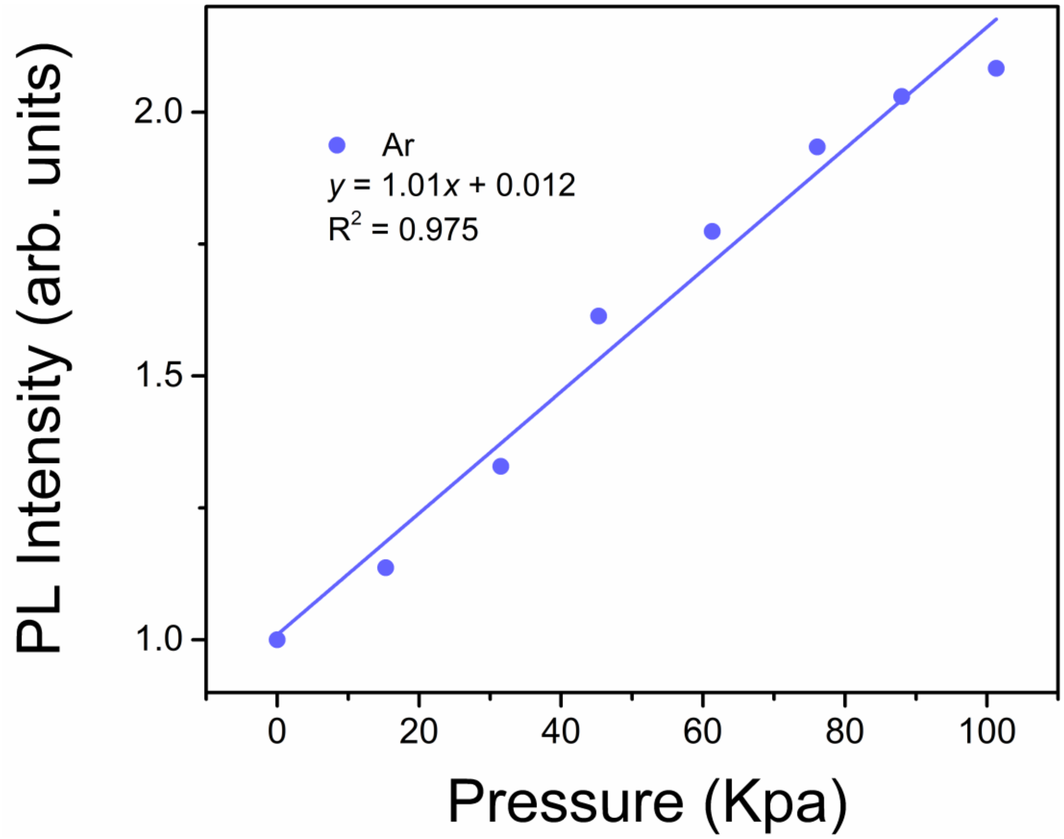

Supplementary Figure 7. The fitted curve for pressure-dependent intensity of FJI-H31(Gd) at 390 nm, Ar.

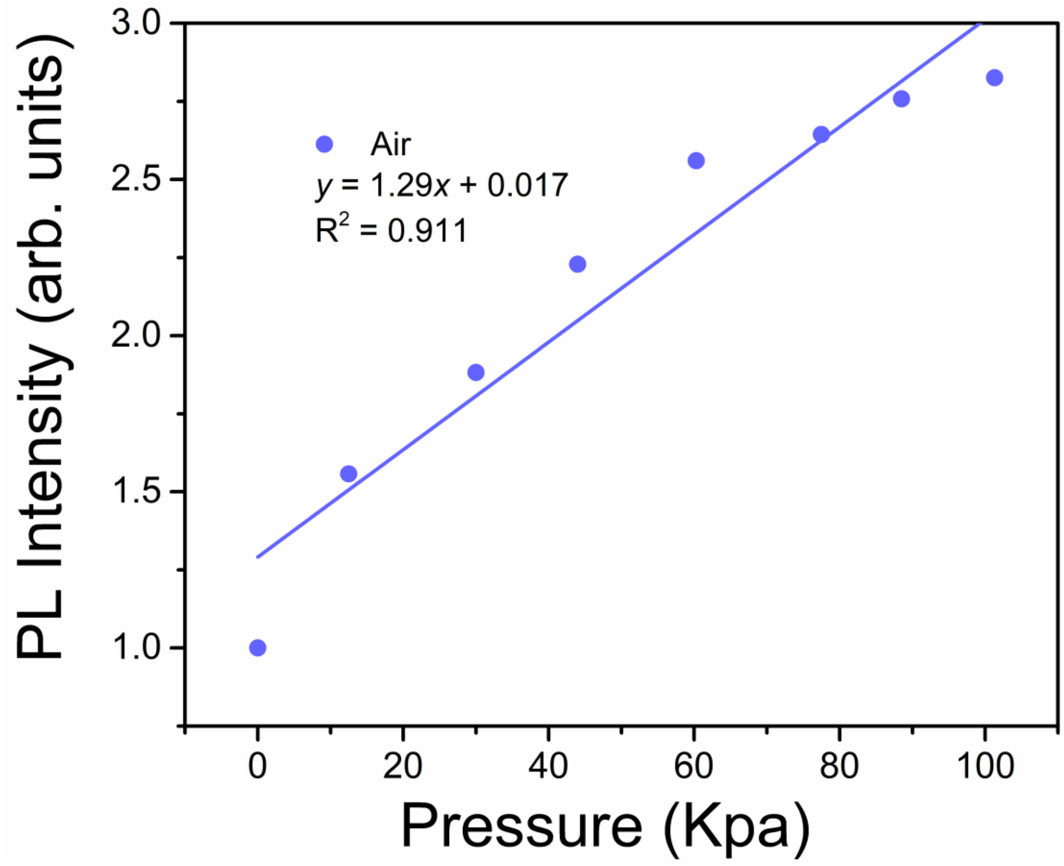

Supplementary Figure 8. The fitted curve for pressure-dependent intensity of FJI-H31(Gd) at 390 nm, Air.

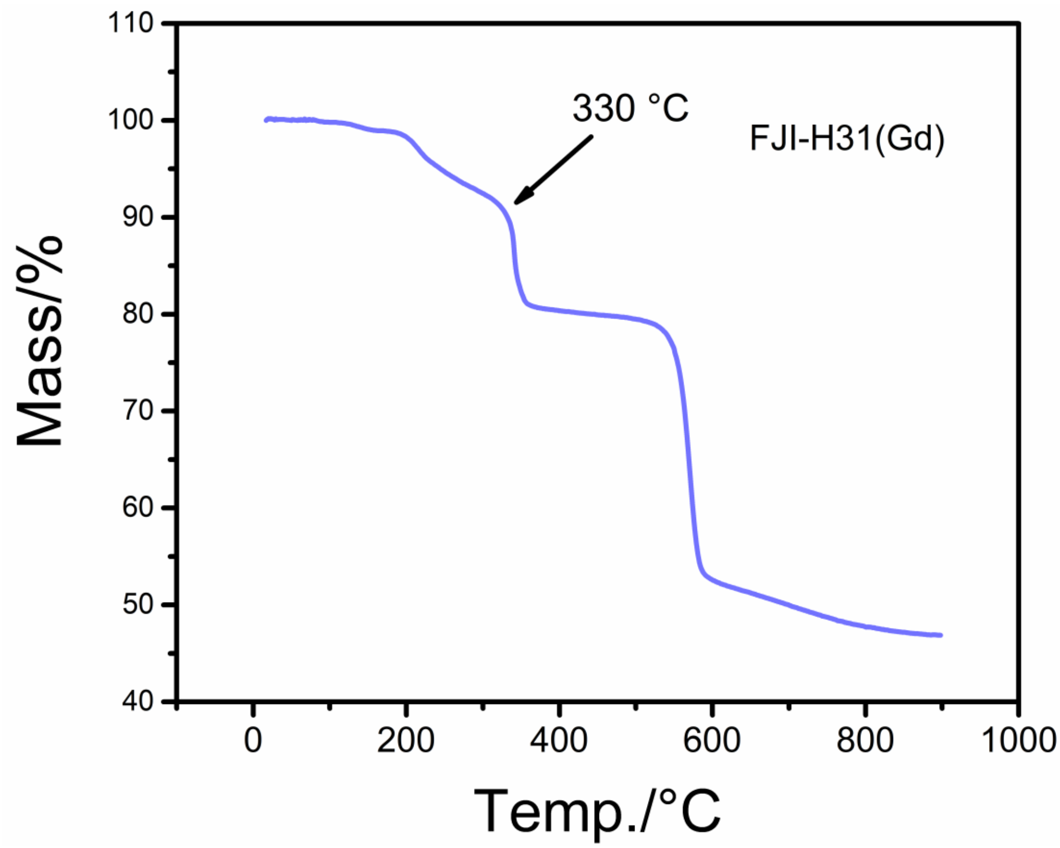

Supplementary Figure 9. The curve of the thermogravimetric analysis of FJI-H31(Gd). FJI-H31 will decompose after 330 ℃.

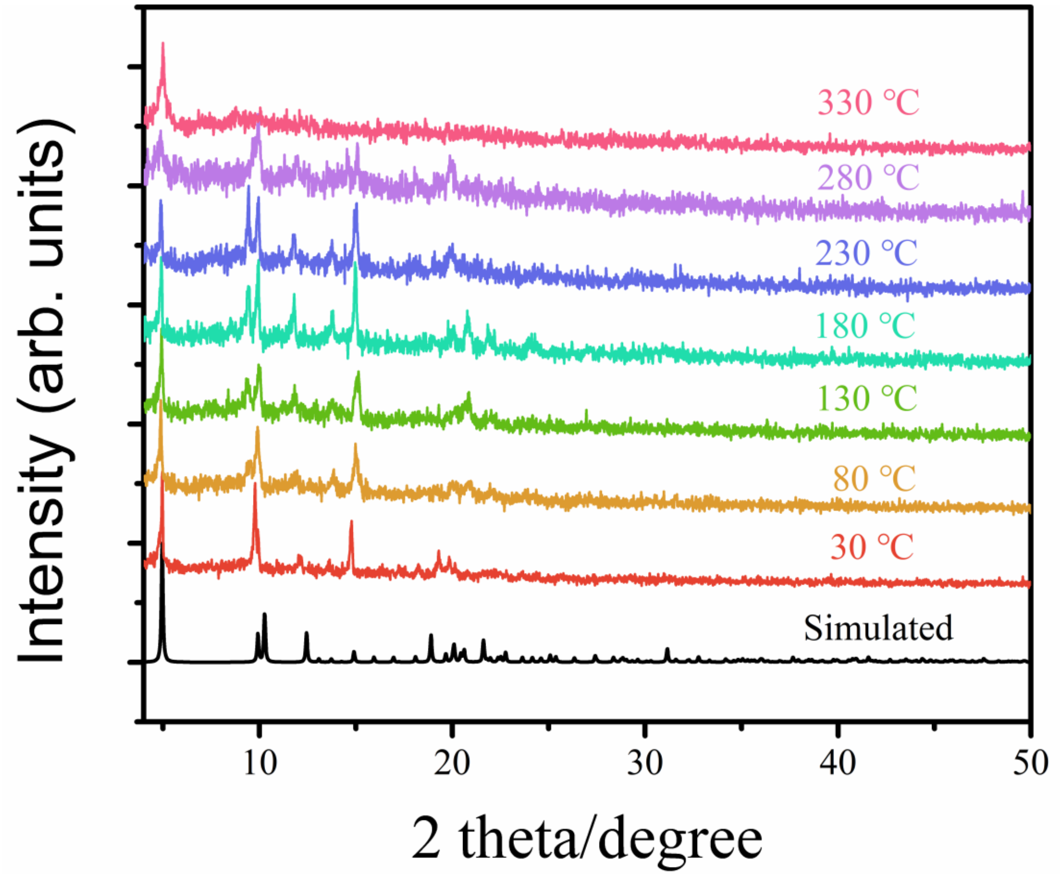

Supplementary Figure 10. Powder XRD patterns of FJI-H31 at different temperatures, compared with simulated FJI-H31.

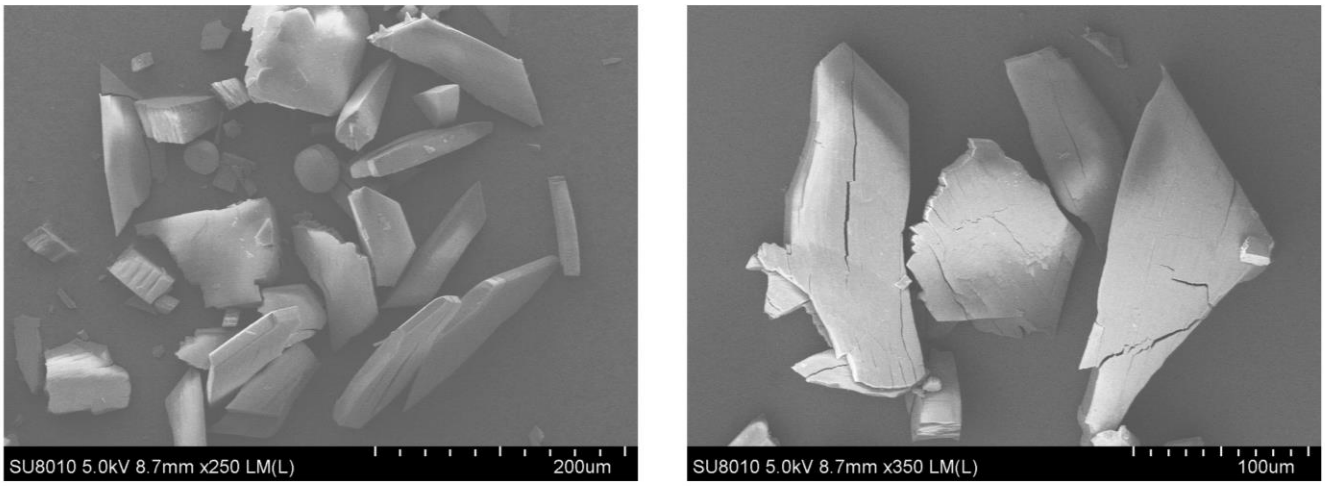

Supplementary Figure 11. SEM images of FJI-H31 before gas pressure sensing.

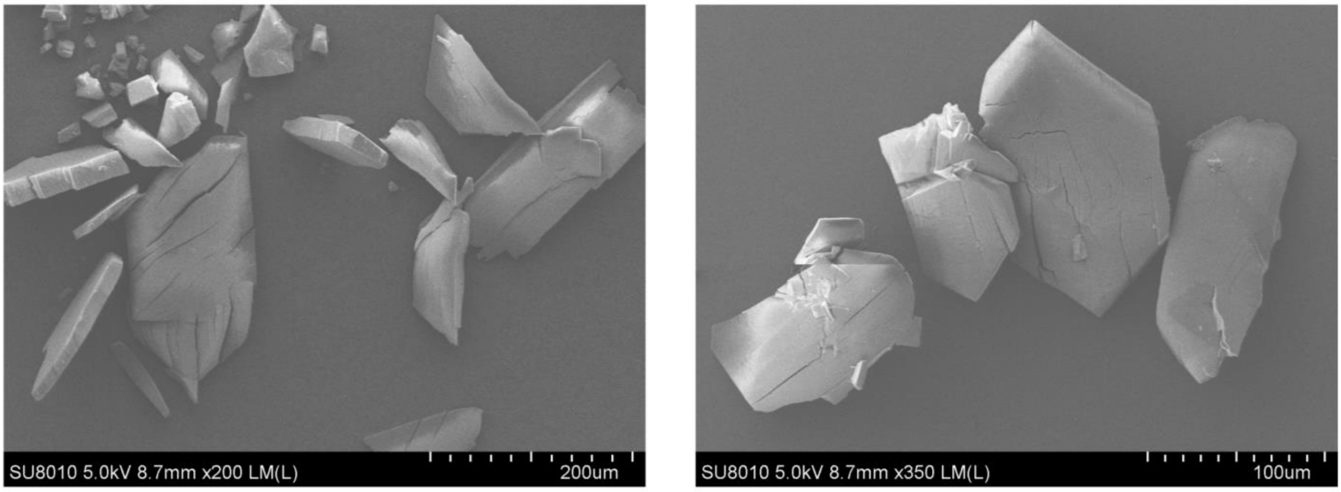

Supplementary Figure 12. SEM images of FJI-H31 after gas pressure sensing.

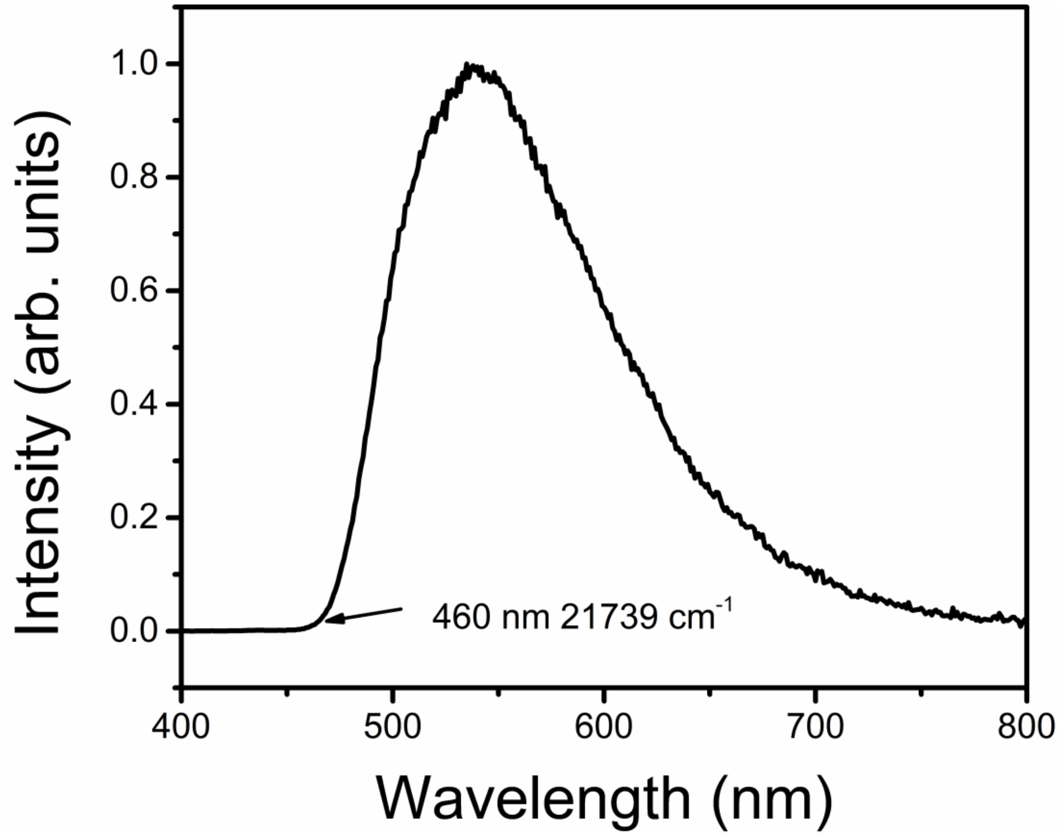

Supplementary Figure 13. Phosphorescence spectrum of FJI-H31(Gd) at 77 K.

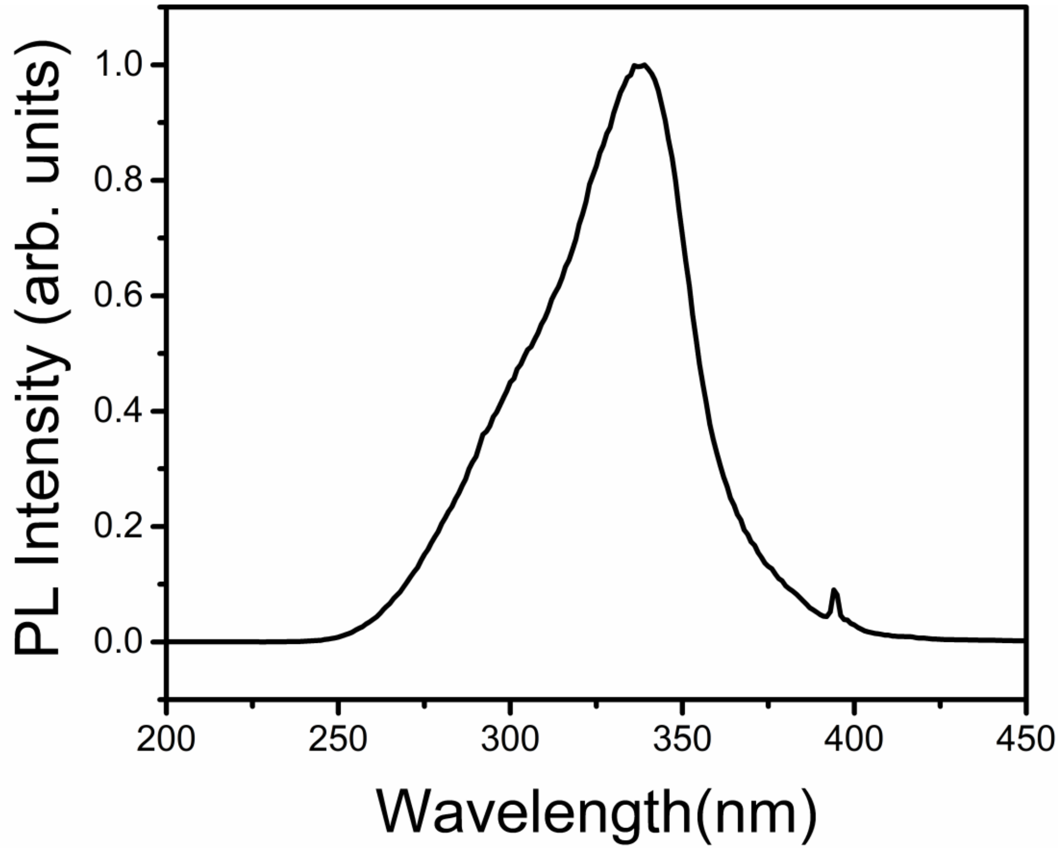

Supplementary Figure 14. Excitation spectrum of FJI-H31(Eu), monitored at 614 nm.

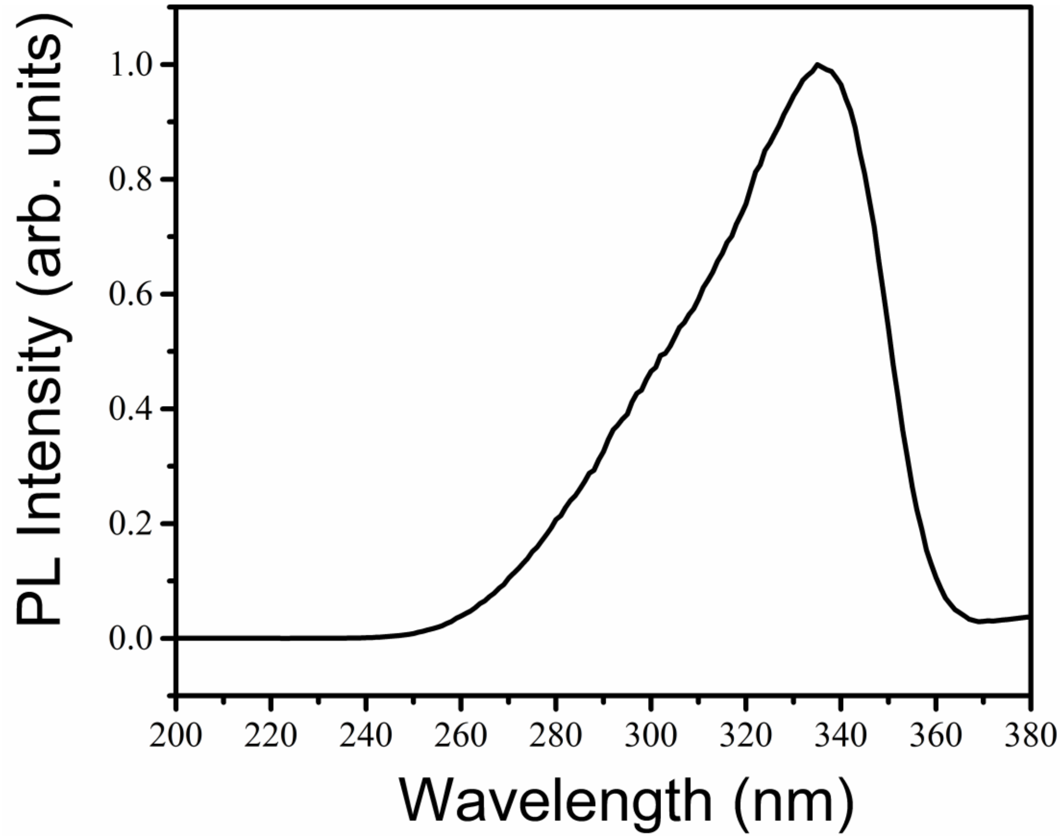

Supplementary Figure 15. Excitation spectrum of FJI-H31(Gd), monitored at 390 nm.

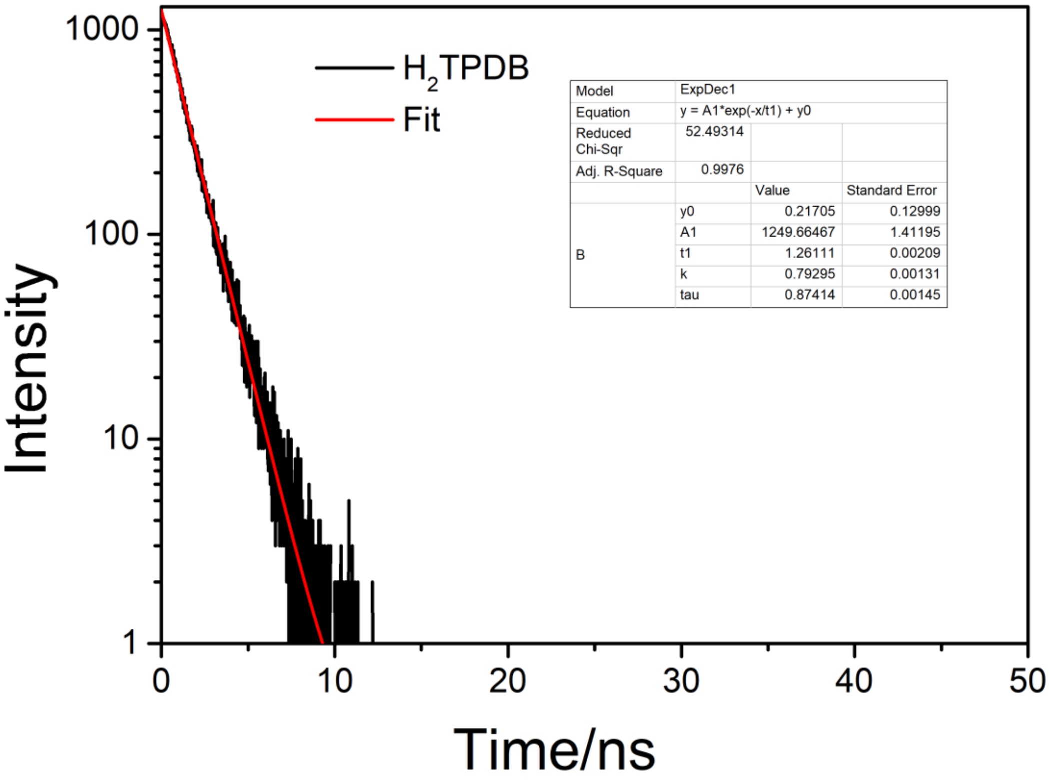

Supplementary Figure 16. The luminescence decay curve of H_2TPDB at 390 nm.

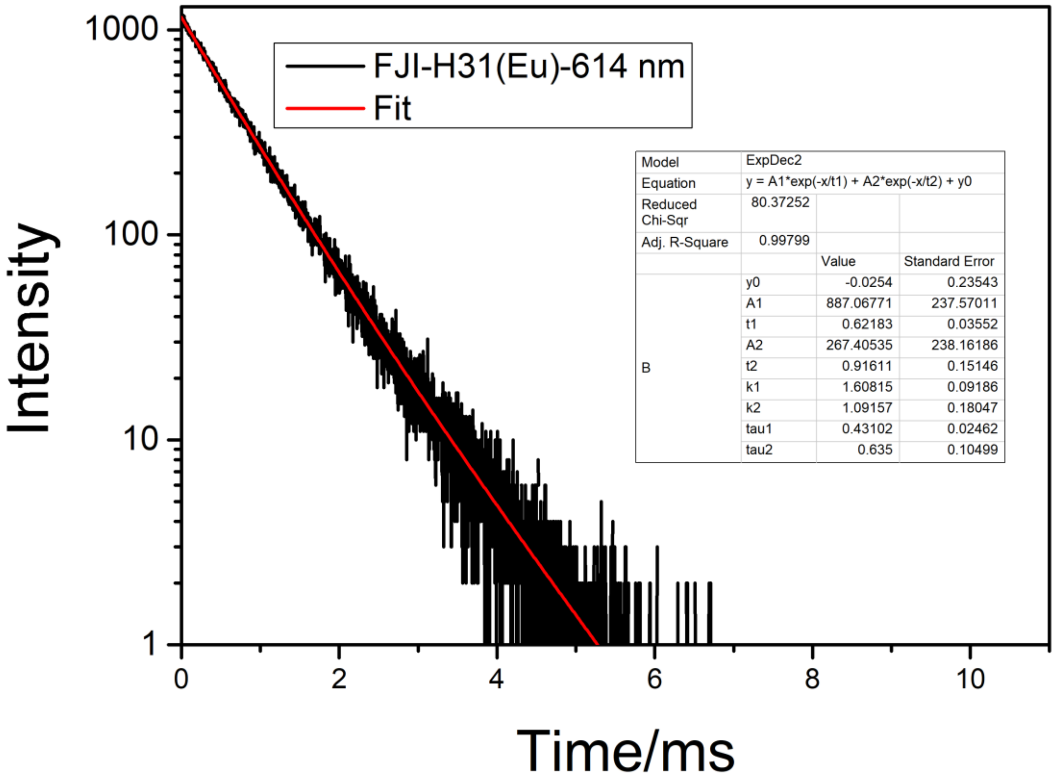

Supplementary Figure 17. The luminescence decay curve of FJI-H31(Eu) at 614 nm.

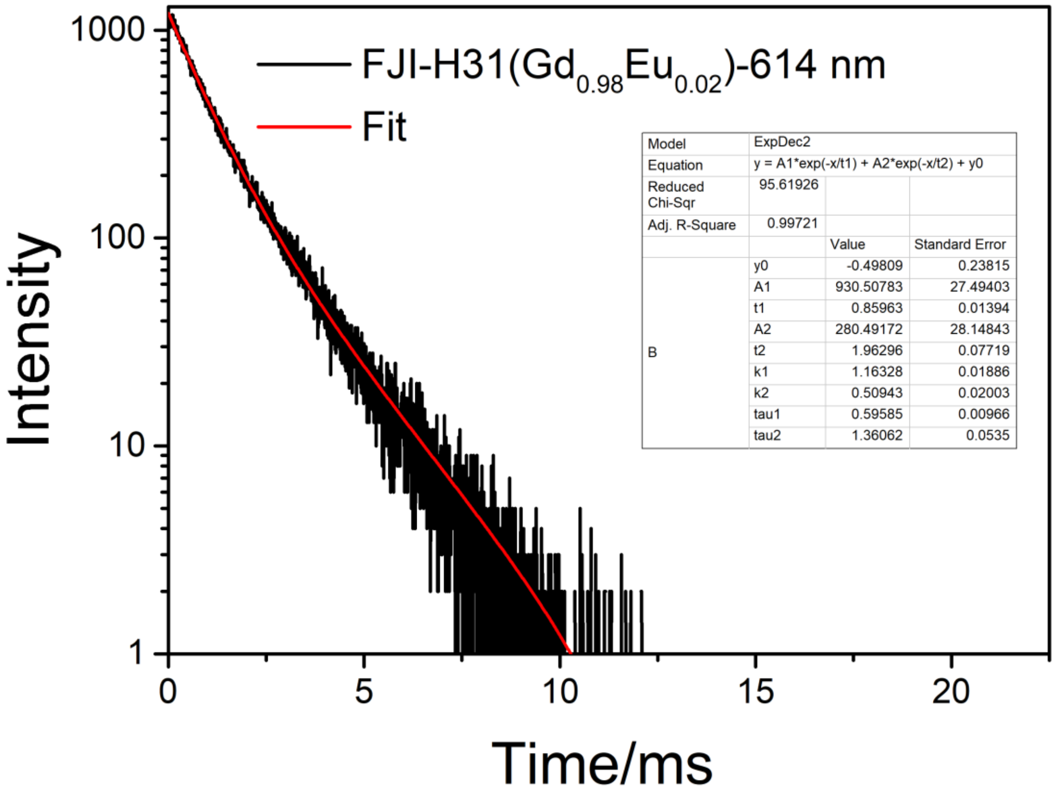

Supplementary Figure 18. The luminescence decay curve of FJI-H31($Gd_{0.98}Eu_{0.02}$) at 614 nm.

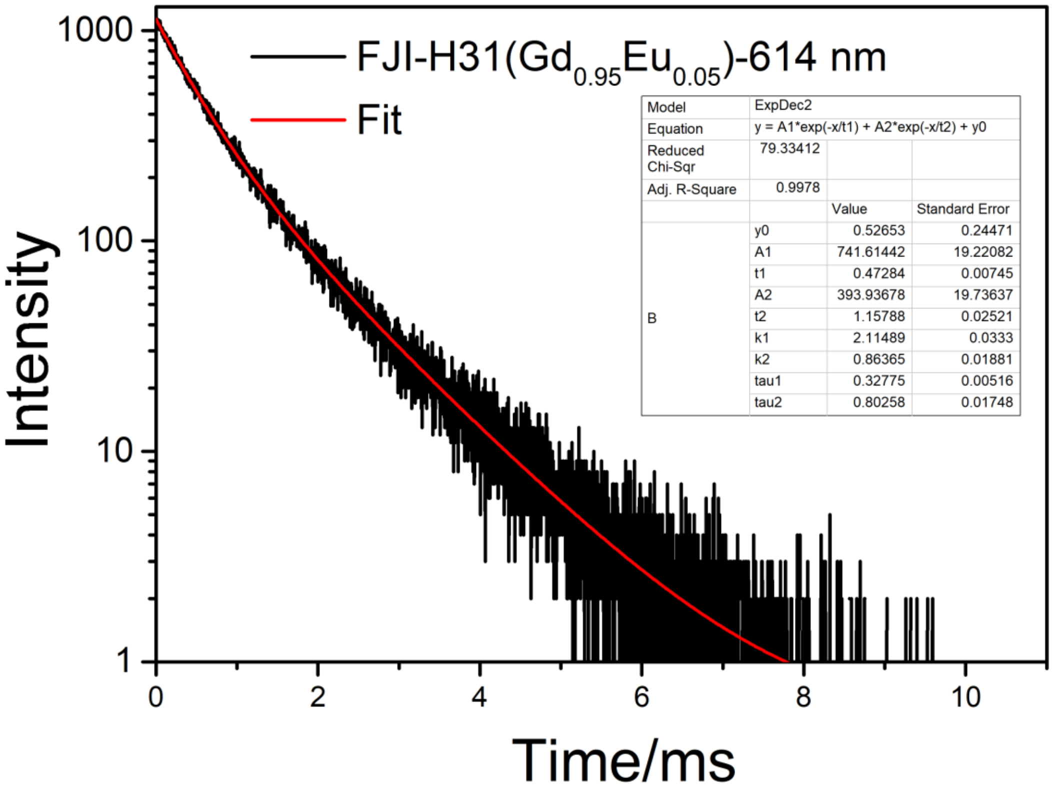

Supplementary Figure 19. The luminescence decay curves of FJI-H31($Gd_{0.95}Eu_{0.05}$) at 614 nm.

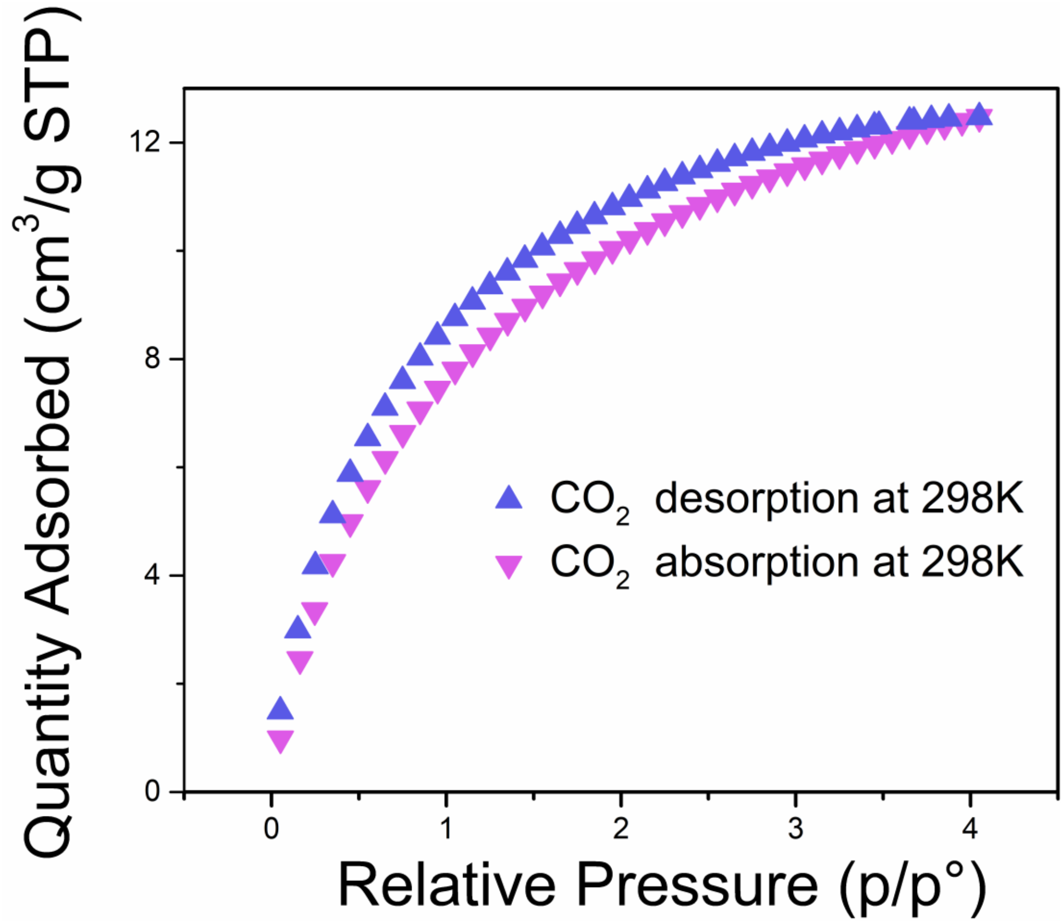

Supplementary Figure 20. The CO_2 adsorption-desorption isotherms of FJI-H31(Gd) at 298K.

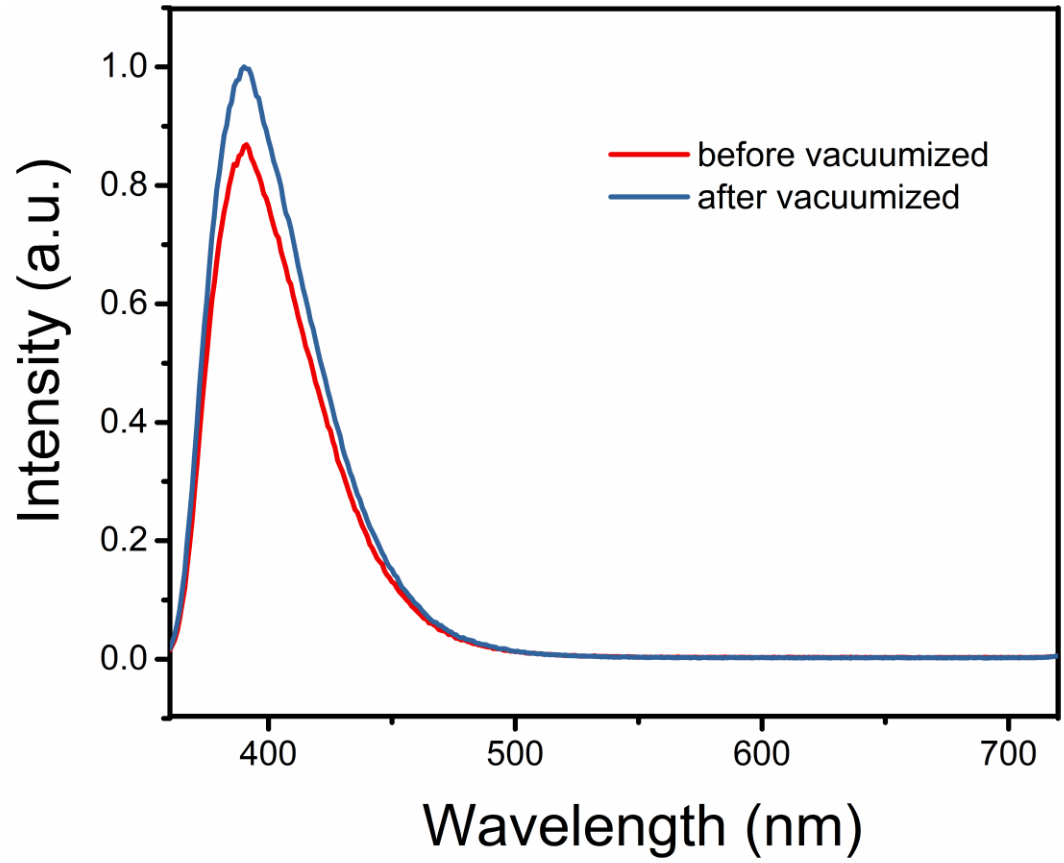

Supplementary Figure 21. Emission spectra of H_2TPDB before and after vacuumized, excited at 338 nm.

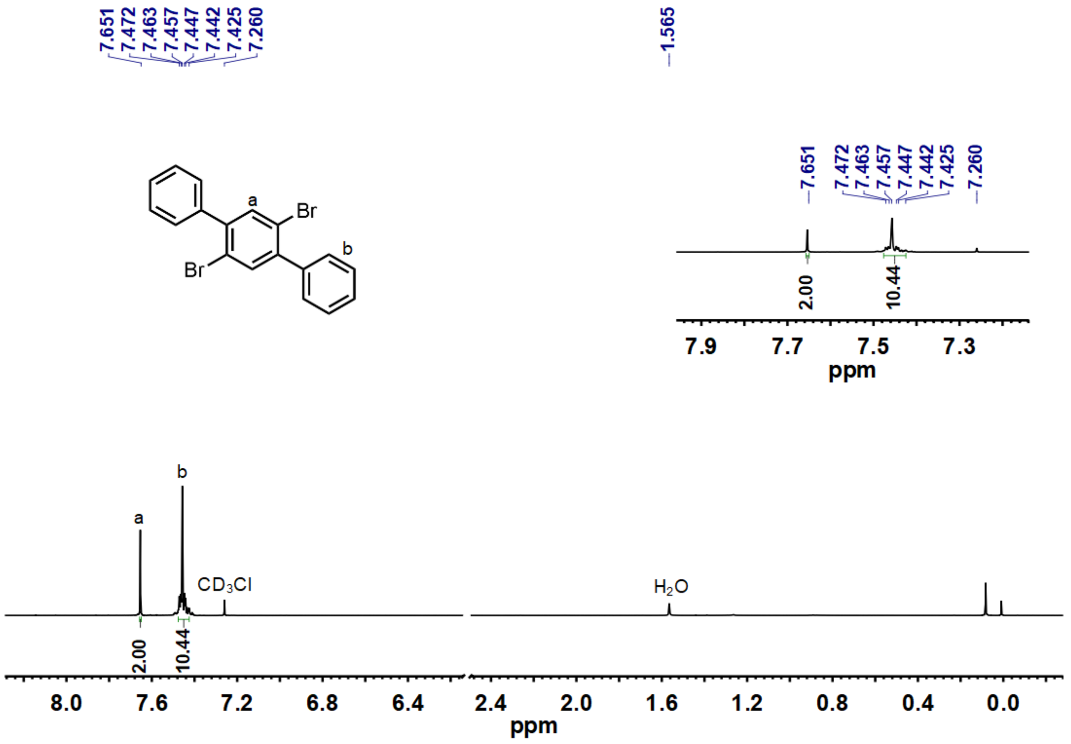

Supplementary Figure 22. The ^{1}H-NMR spectrum of 2',5'-dibromo-1,1':4',1''-terphenyl (400 MHz, $CDCl_3$, 298 K).

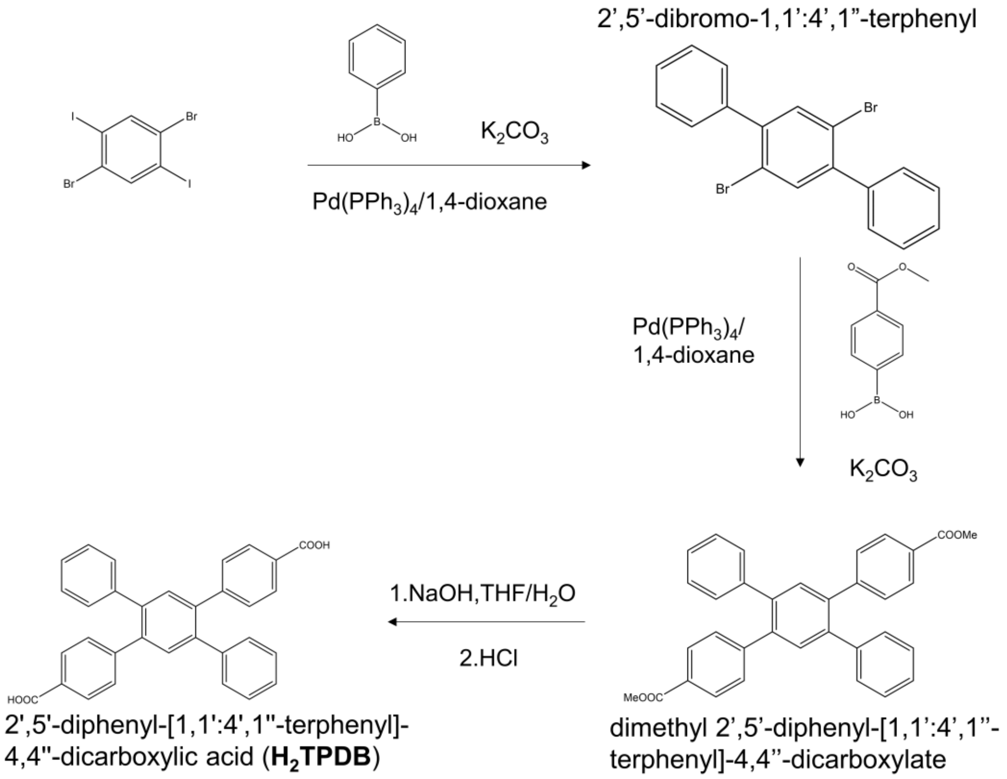

Supplementary Figure 23. Synthesis scheme of the ligand H_2TPDB. Firstly, synthesis of 2’,5’-dibromo-1,1’:4’,1”-terphenyl by 1,4-dibromo-2,5-diiodobenzene and phenylboronic acid; then this product was mixed with (4-(methoxycarbonyl)phenyl) boronic acid to synthesize dimethyl 2’,5’-diphenyl-[1,1’:4’,1’’-terphenyl]-4,4’’-dicarboxylate; finally, the carboxylic ester was hydrolyzed to get the target product H_2TPDB.

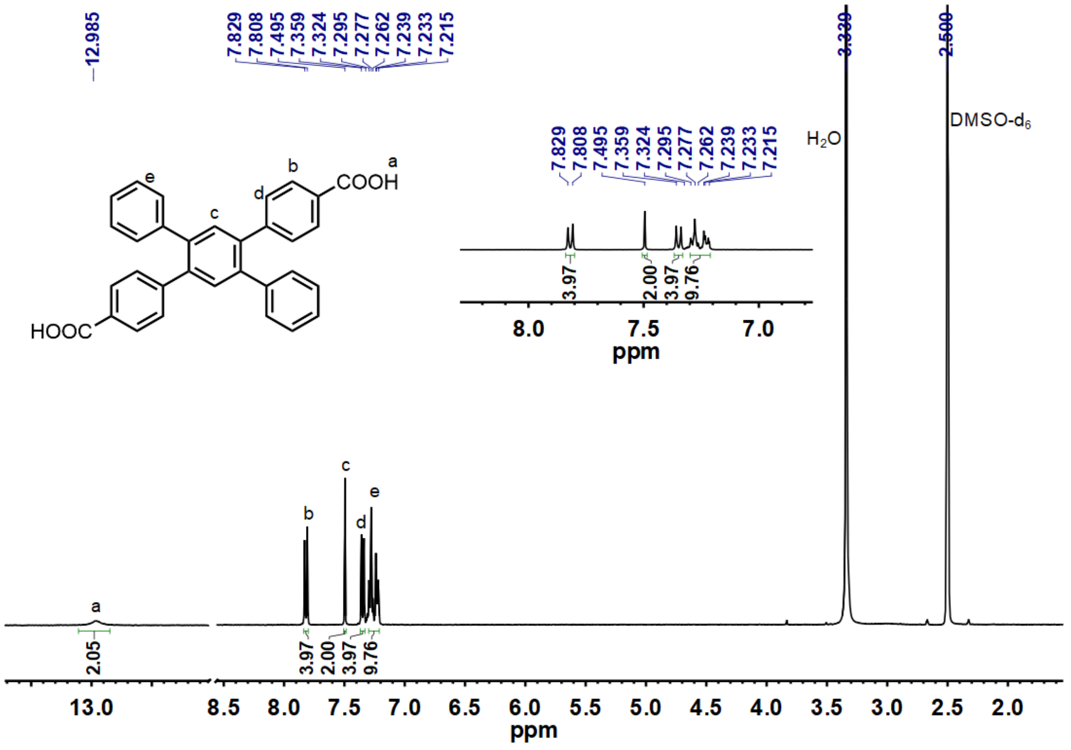

Supplementary Figure 24. The ^{1}H-NMR spectrum of H_2TPDB (400 MHz, DMSO-D_6, 298 K).

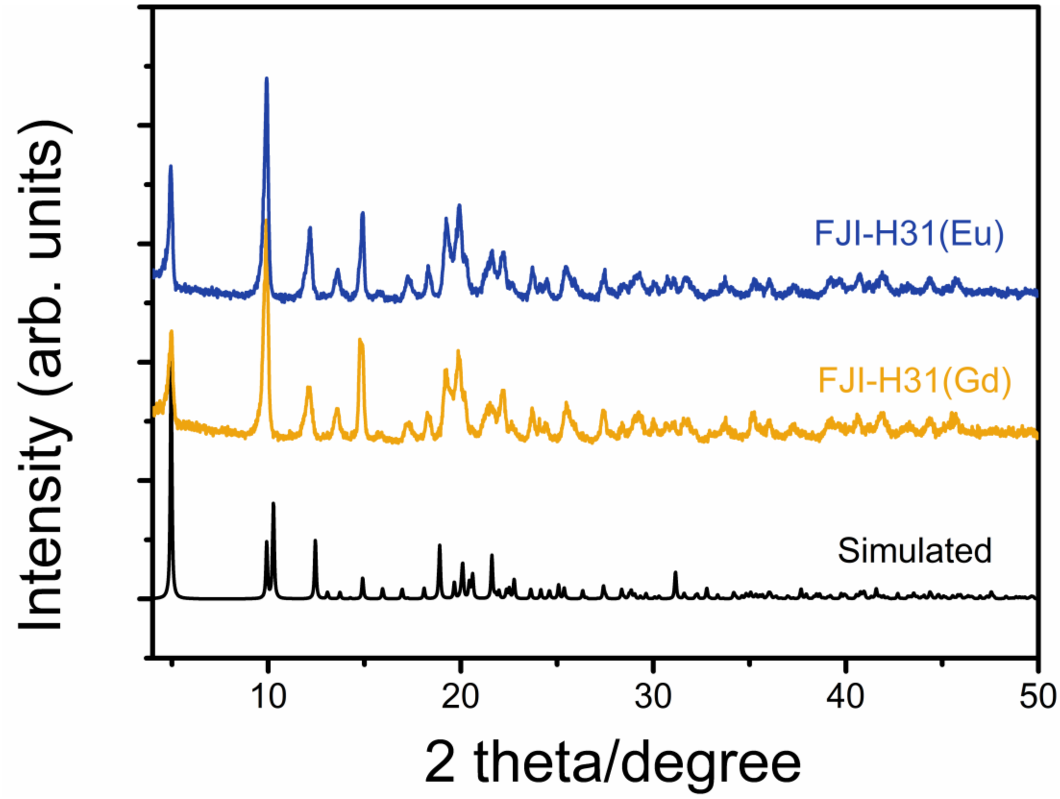

Supplementary Figure 25. Powder XRD patterns of synthesized FJI-H31(Gd) and FJI-H31(Eu).

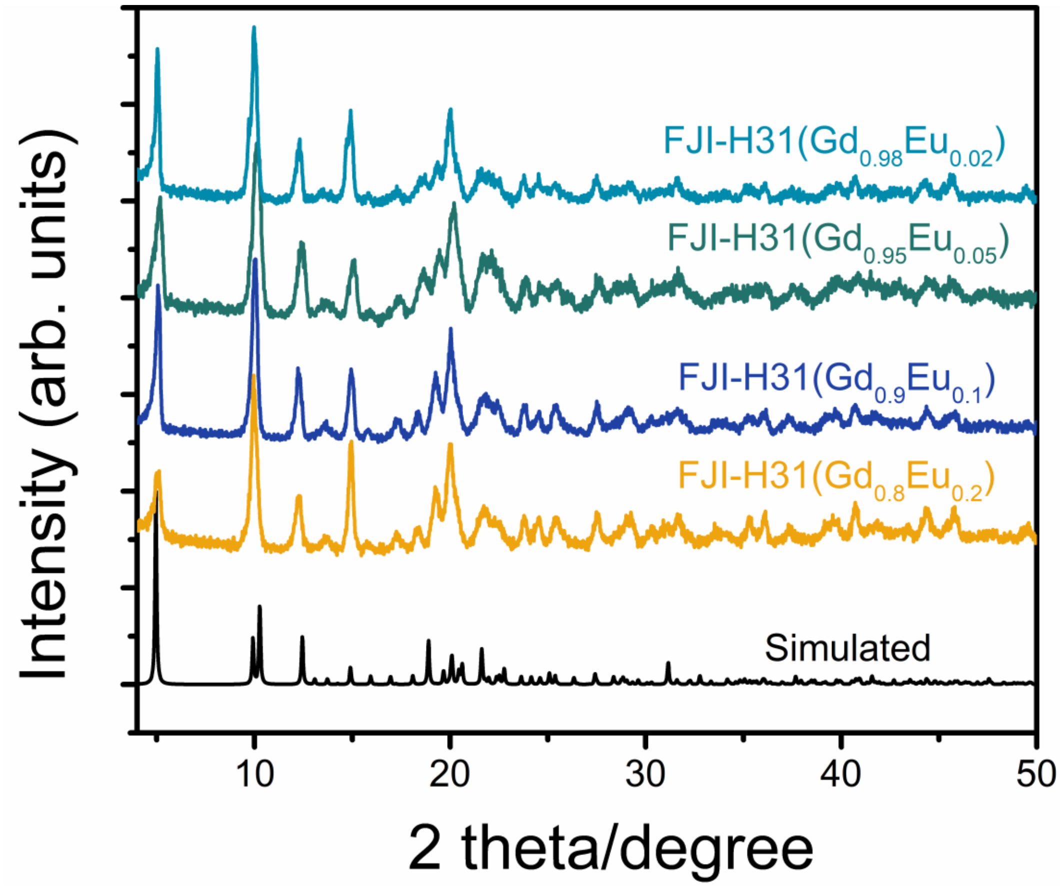

Supplementary Figure 26. Powder XRD patterns of samples with different ratios of Gd^{3+}/Eu^{3+}.

Supplementary Table 1. Crystal data and structure refinement parameters for FJI-H31(Gd) and FJI-H31(Eu).

Empirical formula	$C_{38}H_{38}GdN_2O_8$	$C_{38}H_{38}EuN_2O_8$
Formula weight	807.95	802.66
Crystal system	monoclinic	monoclinic
Space group	*I*2/a	*I*2/a
a/Å	10.4918(2)	10.5092(2)
b/Å	8.87060(10)	8.87120(10)
c/Å	35.7670(4)	35.7831(4)
α/°	90	90
β/°	95.0770(10)	95.0260(10)
γ/°	90	90
Volume/Å^3	3315.72(8)	3323.20(8)
Z	4	4
ρ_{calc}g/cm^3	1.619	1.595
μ/mm^{-1}	13.417	13.974
F(000)	1632.0	1611.0
Reflections collected	15804	15546
Independent reflections	3205 [R_{int} = 0.0439, R_{sigma} = 0.0247]	3224 [R_{int} = 0.0364, R_{sigma} = 0.0233]
Goodness-of-fit on F^2	1.059	1.098
Final R indexes [I>=2σ (I)]	R_1 = 0.0349, wR_2 = 0.0919	R_1 = 0.0373, wR_2 = 0.1040
Final R indexes [all data]	R_1 = 0.0356, wR_2 = 0.0927	R_1 = 0.0381, wR_2 = 0.1054

CCDC 2041736 and 2041737 contains the supplementary crystallographic data for this paper. These data can be obtained free of charge from The Cambridge Crystallographic Data Centre via www.ccdc.cam.ac.uk/structures

Supplementary Table 2. The original ratios of different lanthanide metal salts and the corresponding ICP results.

	**Experimental**		**ICP Results**	
	Gd(y)	Eu(x)	Gd(y)	Eu(x)
FJI-H31($Gd_{0.8}Eu_{0.2}$)	0.8	0.2	0.7747	0.2253
FJI-H31($Gd_{0.9}Eu_{0.1}$)	0.9	0.1	0.8872	0.1128
FJI-H31($Gd_{0.95}Eu_{0.05}$)	0.95	0.05	0.9414	0.0586
FJI-H31($Gd_{0.98}Eu_{0.02}$)	0.98	0.02	0.9747	0.0253
FJI-H31($Gd_{0.995}Eu_{0.005}$)	0.995	0.005	0.9922	0.0078

Supplementary Table 3. The lifetimes and QYs of H_2TPDB and lanthanide-MOFs with different molar ratios of Gd^{3+} and Eu^{3+}.

<table>
<tr><th>Sample</th><th>τ (390 nm)</th><th>τ (614 nm)</th><th colspan="2">ΦF(%)</th></tr>
<tr><td>H_2TPDB</td><td>1.26 ns</td><td>/</td><td colspan="2">53.29</td></tr>
<tr><td>FJI-H31(Gd)</td><td>/</td><td>/</td><td colspan="2">3.46</td></tr>
<tr><td rowspan="2">FJI-H31($Gd_{0.98}Eu_{0.02}$)</td><td rowspan="2">/</td><td rowspan="2">1.31 ms</td><td colspan="2">25.34</td></tr>
<tr><td>ligand/11.65</td><td>Eu/13.69</td></tr>
<tr><td rowspan="2">FJI-H31($Gd_{0.95}Eu_{0.05}$)</td><td rowspan="2">/</td><td rowspan="2">0.86 ms</td><td colspan="2">27.85</td></tr>
<tr><td>ligand/11.65</td><td>Eu/13.69</td></tr>
<tr><td>FJI-H31(Eu)</td><td></td><td>0.71 ms</td><td colspan="2">20.45</td></tr>
</table>

For FJI-H31(Eu)

Alert level B

```
PLAT420_ALERT_2_B D-H Without Acceptor       O3D      --H3DA    .    Please Check
PLAT420_ALERT_2_B D-H Without Acceptor       O3D      --H3DB    .    Please Check
```

Response: This problem may arise from the disordered nature of O3 and H3 atoms.

For FJI-H31(Gd)

Alert level B

```
PLAT420_ALERT_2_B D-H Without Acceptor       O2       --H2A     .    Please Check
PLAT420_ALERT_2_B D-H Without Acceptor       O2       --H2B     .    Please Check
```

Response: This problem may arise from the disordered nature of O2 and H2 atoms.
